# Nitrogen uptake rates and phytoplankton composition across contrasting North Atlantic Ocean coastal regimes north and south of Cape Hatteras

**DOI:** 10.3389/fmicb.2024.1380179

**Published:** 2024-05-09

**Authors:** Yifan Zhu, Margaret R. Mulholland, Peter W. Bernhardt, Aimee Renee Neeley, Brittany Widner, Alfonso Macías Tapia, Michael A. Echevarria

**Affiliations:** ^1^Department of Ocean and Earth Sciences, Old Dominion University, Norfolk, VA, United States; ^2^Department of Marine Sciences, University of Connecticut, Groton, CT, United States; ^3^NASA Goddard Space Flight Center, Greenbelt, MD, United States; ^4^Office of Education, National Oceanic and Atmospheric Administration, Silver Spring, MD, United States

**Keywords:** nitrogen uptake using ^15^N isotopes, phytoplankton community composition, cyanate, Cape Hatteras, Mid Atlantic Bight, South Atlantic Bight, Gulf Stream

## Abstract

Understanding nitrogen (N) uptake rates respect to nutrient availability and the biogeography of phytoplankton communities is crucial for untangling the complexities of marine ecosystems and the physical, biological, and chemical forces shaping them. In the summer of 2016, we conducted measurements of bulk microbial uptake rates for six ^15^N-labeled substrates: nitrate, nitrite, ammonium, urea, cyanate, and dissolve free amino acids across distinct marine provinces, including the continental shelf of the Mid-and South Atlantic Bights (MAB and SAB), the Slope Sea, and the Gulf Stream, marking the first instance of simultaneously measuring six different N uptake rates in this dynamic region. Total measured N uptake rates were lowest in the Gulf Stream followed by the SAB. Notably, the MAB exhibited significantly higher N uptake rates compared to the SAB, likely due to the excess levels of pre-existing phosphorus present in the MAB. Together, urea and nitrate uptake contributed approximately 50% of the total N uptake across the study region. Although cyanate uptake rates were consistently low, they accounted for up to 11% of the total measured N uptake at some Gulf Stream stations. Phytoplankton groups were identified based on specific pigment markers, revealing a dominance of diatoms in the shelf community, while *Synechococcus*, *Prochlorococcus*, and pico-eukaryotes dominated in oligotrophic Gulf Stream waters. The reported uptake rates in this study were mostly in agreement with previous studies conducted in coastal waters of the North Atlantic Ocean. This study suggests there are distinct regional patterns of N uptake in this physically dynamic region, correlating with nutrient availability and phytoplankton community composition. These findings contribute valuable insights into the intricate interplay of biological and chemical factors shaping N dynamics in disparate marine ecosystems.

## 1 Introduction

It has been estimated that the ocean has taken up nearly 25% of the global fossil fuel CO_2_ emissions between 1960–2021 (Friedlingstein et al., 2023). Marine phytoplankton, the ocean’s “invisible forest,” play a critical role in this process through the photosynthesis-respiration redox couple they mediate and their potential to facilitate carbon export to the deep ocean (Falkowski, 2002). Nitrogen (N) is at the heart of marine primary production because its availability often limits the capacity and rate of primary productivity in most of the world’s oceans (Falkowski, 1997; Moore et al., 2013), although iron (Fe) and phosphorus (P) can also limit or co-limit phytoplankton productivity in some regions (Duhamel et al., 2021; Browning and Moore, 2023). Among the various N species present in marine environments, ammonium (NH_4_^+^) is thought to be the preferred N source for most phytoplankton groups (Dortch et al., 1991; Harrison et al., 1996), and turns over rapidly in the euphotic zone, typically on timescales of minutes to hours (Fuhrman et al., 1988; Sunda and Hardison, 2007). Ammonium is thought to support up to 95% of primary production (“regenerated”) in the oligotrophic oceans (e.g., Raimbault et al., 1999; Clark et al., 2008). Nitrate (NO_3_^−^) is typically depleted in surface waters, although it is present at high concentrations in sub-euphotic waters, vertical mixing processes, e.g., upwelling and diffusion, are required to inject NO_3_^−^ into the euphotic zone where it can stimulate (“new”) productivity (Dugdale, 1967; Lewis et al., 1986; Falkowski et al., 1998). In recent years, field studies and physiological and genomic evidence suggest that marine microbes can take up and metabolize a wider variety of N compounds than previously thought, including many organic compounds (Zubkov et al., 2003; Bronk et al., 2007; Moschonas et al., 2017; Kang et al., 2021), further complicating our view of the marine N cycle.

Cyanate, arguably the simplest organic compound, has recently emerged as a marine N cycle intermediate that is bioavailable to cyanobacteria (e.g., Kamennaya et al., 2008; Sato et al., 2022) and other auto-and heterotrophic microbes (e.g., Hu et al., 2014; Zhuang et al., 2015; Widner and Mulholland, 2017; Zhu et al., 2023). The uptake of cyanate accounted for up to 10% of total measured N uptake in the oligotrophic Mid-Atlantic Bight, the Gulf of Maine, and the Eastern Tropical South Pacific Ocean (Widner and Mulholland, 2017; Widner et al., 2018), suggesting it is an overlooked N cycle component in the coastal and open oceans. Furthermore, vertical distributions of cyanate suggest it is an intermediate in the regeneration of particulate organic N (PON), exhibiting a nutrient-like profile, not unlike that of nitrite (Widner and Mulholland, 2017; Widner et al., 2018).

Urea, another simple organic compound that represents only a minor fraction of the total dissolved organic N (DON) pool in most estuarine and coastal waters, with concentrations generally less than 1 μmol N L^−1^ (Sipler and Bronk, 2015), has long been known to fuel primary productivity (Mulholland and Lomas, 2008, and references therein). Unlike cyanate, urea assimilation by phytoplankton can contribute 50% or more of the total N taken up by planktonic communities in some systems (see the review in Solomon et al., 2010), suggesting urea, like ammonium, turns over rapidly (Price and Harrison, 1988). Urea pathways are found in nearly all genomes of marine cyanobacteria (Scanlan et al., 2009) and distinct from those in eukaryotic phytoplankton (Solomon et al., 2010; Wang et al., 2023).

Similar to cyanate and urea, phytoplankton compete with heterotrophic microbes for amino acid nitrogen (Kirchman et al., 1994). Up to 51–82% of phytoplankton biomass is comprised of proteins and amino acids (Nguyen and Harvey, 1997), so it is not surprising that these compounds are released during phytoplankton metabolism and decomposition (Sarmento et al., 2013), or as a result of “sloppy feeding” during grazing (Fuhrman, 1987). Like other organic matter degradation products, e.g., urea, ammonium, and cyanate, amino acids comprise only a small fraction (1.2–12.5%) of the largely uncharacterized dissolved organic nitrogen pool but turn over rapidly (e.g., minutes) (Sipler and Bronk, 2015). Fuhrman (1987) indicated that the release and uptake of free amino acids by phytoplankton were tightly coupled during a 20-month observational study along the mid-Atlantic continental shelf. Another study indicated that amino acid assimilation rates by phytoplankton varied across salinity gradients (Mulholland et al., 1998). The assimilation of amino acids can be facilitated through the activity of cell-surface enzymes that oxidize amino acids producing ammonium (Palenik and Morel, 1990; Mulholland et al., 1998, 2002). Transcriptomes of diatoms, pelagophytes, and dinoflagellates indicate that these groups possess amino acid transporters, suggesting direct assimilation of amino acids by phytoplankton (Frischkorn et al., 2014; Alexander et al., 2015a; Wohlrab et al., 2018).

In waters influenced by shelf processes, marine organisms live in environments where the resources on which they depend fluctuate spatially and temporally (Polis et al., 1997). During winter months, wind-driven mixing can entrain nitrate from depth into surface waters whereas, in summer, strong stratification impedes nitrate resupply from depth, often leaving surface waters depleted in fixed N. In addition, perturbations associated with hydrographic features such as ocean currents and fronts (d’Ovidio et al., 2010; Kuhn et al., 2019), as well as shorter-lived mesoscale eddies and meteorological events all contribute to the dynamic nutrient environment (Polis et al., 1997; McGillicuddy, 2016). Hydrodynamic changes can trigger responses at the cellular level, as phytoplankton modify their eco-physiological traits to acclimate to a fluctuating environment, e.g., differential nutrient uptake strategies, including resource storage, high resource use efficiency during periods of scarcity, and dormant life stages (Yang et al., 2010; Litchman et al., 2015; Lin et al., 2016). These traits vary in space and time and can result in distinct temporal and regional distributions of phytoplankton assemblages (Sunda et al., 2006; Barton et al., 2013b). A typical example might be the seasonal succession from diatoms in late winter/early spring to dinoflagellates (or haptophytes/pelagophytes) in summer in certain regions such as mid-to high-latitude surface waters (e.g., Hinder et al., 2012). Diatom blooms deplete inorganic nutrients quickly and fuel the growth of zooplankton, which results in the accumulation of regenerated organic [urea and other DON and dissolved organic phosphorus (DOP)] and inorganic nutrients (e.g., NH_4_^+^) from slopy feeding/excretion by zooplankton, virus lysis, and/or phytoplankton release (Lin et al., 2016). During summer, as nitrate is depleted, the water column becomes stratified, and zooplankton grazing intensifies, the environment becomes less favorable for diatom growth. This sets the stage for a population shift towards phytoplankton groups such as dinoflagellates, whose growth and survival are favored due to their versatile nutrient acquisition strategies, including diel vertical migration, utilization of DON and DOP, cysts production, phagotrophy, and their ability to minimize grazing losses through large cell sizes and the production of toxins and other secondary metabolites (Barton et al., 2013a; Deeds et al., 2014; Lin et al., 2016; Brosnahan et al., 2017). Therefore, investigating the impact of environmental factors on phytoplankton composition and changes in N uptake and understanding the linkages between environmental regime shifts, trait modifications, and community structure is crucial for comprehending dynamic interactions within marine ecosystems.

The western North Atlantic coastal region off the U.S. east coast is a highly productive marine system and an important net sink of atmospheric CO_2_ (Hofmann et al., 2011; Signorini et al., 2013). The Mid-Atlantic Bight (MAB) and South Atlantic Bight (SAB) shelves (delimited as waters less than 200 m in depth) are two coastal subregions separated at Cape Hatteras. The southern coastal MAB is highly influenced by the outflow from the Chesapeake Bay, the southward flow from the northern MAB, and the Slope Sea (Flagg et al., 2002; Todd, 2020); the latter two form a shelf-break frontal zone where mixing is often enhanced and exports of shelf water can occur (Gawarkiewicz et al., 1996; Todd, 2020). In contrast, south of Cape Hatteras, the SAB continental shelf circulation is impacted by shelf water moving north toward Cape Hatteras, and the northward flowing Gulf Stream (Andres et al., 2023). The strong convergence of the alongshore flows at the Hatteras Front can induce cross-shelf exchanges just north of Cape Hatteras due to density instabilities, transporting significant portions of the shelf water to the open ocean (Savidge and Savidge, 2014; Todd, 2020), although there is high temporal and spatial variability in the location of the front due to complex hydrodynamics (Seim et al., 2022). Over the past 40 years, many research programs have been implemented in this region, e.g., the Shelf Edge Exchange Processes experiment (SEEP-II), the Ocean Margins Program, and the Processes driving Exchange At Cape Hatteras (PEACH) studies. However, to date, nutrient dynamics, e.g., nutrient concentrations, uptake of dissolved N, and phytoplankton community composition, in this region have remained poorly understood, in part because previous research was either focused on regional hydrodynamic variability or model simulations of cross-shelf exchanges of carbon and nitrogen. The lateral fluxes of nutrients resulting from the convergence of many different waters masses near Cape Hatteras is thought to play a key role in primary production in this region (e.g., Lohrenz et al., 2002; Friedrichs et al., 2019). Recently, a dinitrogen (N_2_) fixation hotspot was identified in coastal waters near the front (Mulholland et al., 2019; Selden et al., 2021), and the impact of Gulf Stream frontal eddies on ecology was investigated (Gray et al., 2023), underscoring the importance of physical processes in regulating N biogeochemistry in the region.

Here we examined nutrient biogeochemistry with respect to the phytoplankton composition and net community N uptake rates in physically complex coastal waters to the north and south of the Cape Hatteras in the MAB and SAB, Slope Sea, and the Gulf Stream. Our main objectives for this research were to assess: (1) the relative uptake rates of N species, including nitrate, nitrite, ammonium, urea, cyanate, and amino acids, by microbial communities, (2) the distribution and variations in phytoplankton community composition, and (3) the link between the two. We hypothesized that (1) during the summer months, larger cells (e.g., diatoms), pico-eukaryotes, and picocyanobacteria (e.g., *Synechococcus*) would to be prevalent in the continental shelf and Slope Sea regions, whereas picocyanobacteria such as *Prochlorococcus* would dominate the oligotrophic Gulf Stream region (e.g., Fowler et al., 2020; Caracappa et al., 2022; Stevens et al., 2023); (2) the contrasting environmental conditions, e.g., temperature and nutrient levels, across these regions would explain variations in phytoplankton community composition; (3) both nutrient supply and phytoplankton community composition account for discrepancies in the observed N uptake rates (e.g., Lomas and Glibert, 2000; Mulholland and Lomas, 2008; Barton et al., 2013a; Lampe et al., 2019; Inomura et al., 2023).

## 2 Materials and methods

### 2.1 Hydrographic measurement

**Figure 1:**
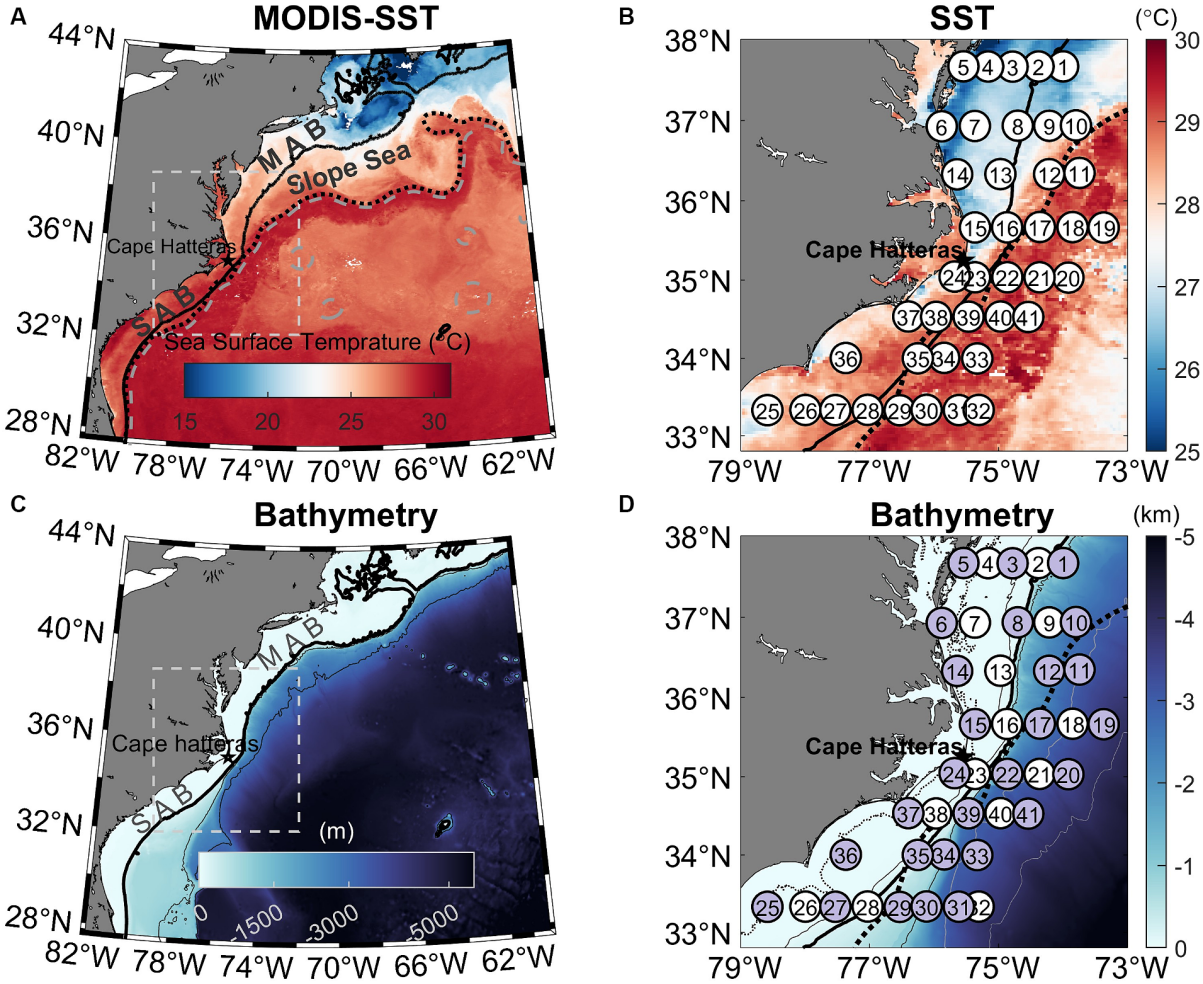
**(A)** Map of the Northwestern Atlantic Ocean showing the 8-day MODIS mean sea surface temperature (SST) from 4–11 August 2016. The white box indicates the study area. The overlaying black dotted and gray dashed lines are SSH contours of 0.2 and 0.5 m, respectively, in which the 0.2 m SSH contour denotes the Gulf Stream Edge (Muglia et al., 2022). **(B)** Zoom-in view of the MODIS SST image highlighting the Gulf Stream and colder Mid-Atlantic Bight (MAB) shelf water and warmer South Atlantic Bight (SAB) shelf water. The white circles represent 41 sampling stations occupied between August 7–17, 2016. **(C)** Bathymetry of the Northwestern Atlantic Ocean, highlighting the study area. **(D)** Zoomed-in view of the study area showing bathymetry across the southern MAB and northern SAB and the cruise transects, in which purple circles represent 27 stations where N uptake experiments using ^15^N tracers were performed. Also marked are 20, 50, 200, 1,000, 3,000, and 4,000 m isobaths in **(D)**. The thick black solid line in all panels mark the 200 m isobaths. The black dotted line in panels **(B)** and **(D)** are SSH contours of 0.2 m.

Standard hydrographic measurements of temperature, salinity, and chlorophyll (Chl) fluorescence were made using a Seabird SBE 911 conductivity-temperature-depth (CTD) unit combined with a fluorometer mounted to a rosette sampler equipped with 24 10 L Teflon-coated Niskin bottles during a cruise from August 7 to 17, 2016, aboard the R/V *Hugh R. Sharp*. The cruise was comprised of eight cross-shelf transects (41 stations in total) that spanned a region between 33.5 and 38° N latitude at approximately 0.5° intervals. Stations were occupied in coastal, continental shelf, and oceanic waters in the MAB and SAB (Figures 1A,B). Station depths (Figures 1C,D) ranged from 10–3,450 m with most stations located on the continental shelf (<200 m) and in the Gulf Stream. Stations were also occupied in waters influenced by the shelf-break jet (200–1,000 m) and in the Slope Sea (>1,000 m). At each station, water for the determination of chemical and biological properties was collected at various depths using Niskin bottles (see below).

### 2.2 Nutrient analyses

Nutrient samples at each depth were collected directly from Niskin bottles through a 0.2 μm capsule filter (Pall Supor^®^) using a peristaltic pump at pressure ≤5 mm Hg. Filtrate was collected directly into two 50 mL sterile polypropylene centrifuge tubes (Falcon^®^) to measure NO_3_^−^, nitrite (NO_2_^−^), orthophosphate (simplified as PO_4_, sum of HPO_4_^2−^ and PO_4_^3−^), and urea concentrations. Filtrate was also pumped into three 2 mL sterile polypropylene tubes for cyanate determinations, two 15 mL OPA-treated polypropylene tubes (Falcon^®^) for NH_4_^+^ analyses, and two 40 mL combusted amber glass vials for analysis of total dissolved free primary amine. To minimize contamination from filtration and between samples, filters and tubing were rinsed thoroughly with site water before sample collection. NO_3_^−^ + NO_2_^−^, NO_2_^−^, PO_4_, and urea samples (duplicates) were stored at 4°C until analysis within 48 h of their collection using a nutrient autoanalyzer (Astoria-Pacific, Inc., United States) according to the manufacturer’s specifications. The method detection limits for NO_3_^−^ + NO_2_^−^, NO_2_^−^, PO_4_, and urea were 0.14 μmol L^−1^, 0.07 μmol L^−1^, 0.03 μmol L^−1^, and 0.08 μmol L^−1^, respectively. NH_4_^+^ samples (duplicates) were kept at 4°C until analysis within 24 h of collection. The concentrations of NH_4_^+^ were measured onboard using the OPA-fluorescence method of Holmes et al. (1999) with a spectrofluorometer. The method detection limit was 10.0 nmol L^−1^. Cyanate samples (triplicates) were stored in liquid nitrogen aboard the ship and at -80°C once samples were returned to the land-based laboratory. Cyanate concentrations were measured by high-performance liquid chromatography (HPLC) using a precolumn fluorescence derivatization method (Widner et al., 2013; Widner and Mulholland, 2017). The method detection limit was 0.4 nmol L^−1^ (Widner et al., 2013). Concentrations of total dissolved free primary amine were measured using the method of Aminot and Kérouel (2006) to estimate ambient dissolved free amino acids (DFAA) concentrations, because the two measurements are generally in agreement (Kirchman et al., 1989). The method detection limit was 4 nmol L^−1^.

### 2.3 Particulate nitrogen measurement

Whole water samples were collected from three target depths at each station: near surface, a depth above the Chl maximum (with majority of them near the bottom of the mixed layer), and at the depth of the Chl maximum. Water from Niskin bottles was drained into individual 10 L carboys and then transported to the ship-board laboratory, where the water samples in each carboy were mixed and sub-samples (0.2–1.4 L) were collected onto pre-combusted GF-75 filters (Whatman^®^, nominal pore size 0.3 μm) in triplicate, for analysis of particulate nitrogen (PN) and carbon (PC) concentrations and the natural abundance of ^15^N and ^13^C. The filters were stored in cryovials and immediately frozen and stored in a freezer at -20°C until analysis. Prior to their analysis, filters were dried at 40°C, pelletized in tin capsules, and analyzed on a Europa 20/20 isotope ratio mass spectrometer equipped with an automated N and C analyzer. The average detection limit of the mass spectrometer was 0.0018 and 0.0005 atom% for ^15^N and ^13^C, respectively; these values were derived based on three times the standard deviation (3 × SD) of the atom% of 12.5 μg N and 100 μg C standards analyzed with each sample run (40 in total).

### 2.4 Short-term nitrogen uptake incubations

Parallel sets of triplicate whole water (0.5–2 L) at the same three target depths mentioned above from 27 stations (Figure 1) were dispensed from the 10 L carboys into acid-cleaned polyethylene terephthalate glycol incubation bottles (Nalgene^™^). Nitrogen uptake incubations were initiated by amending incubation bottles with highly enriched (98–99%) ^15^N-labeled substrates (Cambridge Isotope Laboratories, Inc., United States), including ammonium chloride (^15^NH_4_Cl), potassium nitrate (K^15^NO_3_), potassium nitrite (K^15^NO_2_), and ^15^N-and ^13^C-labeled potassium cyanate (KO^13^C^15^N), urea (^13^CO(^15^NH_2_)_2_), and algal amino acid mixture. The final ^15^N enrichment after tracer amendments were mostly 5–50%, but some exceeded 50% so should be considered as potential uptake rates. After the tracer additions, bottles were placed in deck-board incubators with underway surface seawater flowing through to achieve near *in-situ* temperatures and covered with neutral density screens to reduce incident light to approximately 55, 28, 14% of the ambient light levels approximating those observed at the depth of sample collection. The incubations were generally accomplished during daylight hours but there were 7 stations at which incubations were conducted at nighttime. For nighttime incubations, incubators were covered with an opaque tarp after sunset until just before sunrise to prevent the ship’s deck lights from affecting the incubations. After 2–3 h, incubations were terminated by filtration through combusted GF-75 filters at pressure ≤5 mm Hg. Filters were placed in sterile cryovials and stored at -20°C until their analysis. Prior to analysis, samples were dried at 40°C and then pelletized into tin disks. Final PN concentrations and the corresponding atom% enrichment of the PN pool were measured on a Europa 20/20 isotope ratio mass spectrometer as mentioned above. In this study, heterotrophic contributions to the total measured N uptake were not estimated.

### 2.5 Nitrogen uptake rate calculations

Absolute nitrogen uptake (or “transport”) rates were calculated using a mixing model (Mulholland et al., 2006; Eq. 1).


(1) $$N\;15\;\mathrm{uptake rate}=\frac{{\left(\mathrm{atom}\%\;\;\mathrm{PN}\right)}_{\mathrm{final}}-{\left(\mathrm{atom}\%\;\mathrm{PN}\right)}_{\mathrm{initial}}}{{\left(\mathrm{atom}\%\;N\;\mathrm{source pool}-\mathrm{atom}\%\;\mathrm{PN}\right)}_{\mathrm{inital}}}\times \frac{\left[\mathrm{PN}\right]\;}{\mathrm{time}}$$

where ${\left(\mathrm{atom}\%\;\;\mathrm{PN}\right)}_{\mathrm{initial}}$ and ${\left(\mathrm{atom}\%\;\;\mathrm{PN}\right)}_{\mathrm{final}}$ represent the ^15^N isotopic composition of the particulate pool at the initial and final time points of the incubation period; atom%Nsource pool is the ^15^N isotopic enrichment of the dissolved nitrogen pool after the tracer addition; [PN] represents the concentration of the particulate nitrogen pool; here, we used the average value of the initial and final PN concentrations. Post-incubation measurements confirmed that substrate consumption over the incubation period while significant, was less than 14% (^15^NO_3_^−^), 18% (^15^NO_2_^−^), 27% (^15^NH_4_^+^), 10% (urea), 4% (cyanate), and 25% (amino acid) of initial addition. If 50% of the added substrates were taken up, which occurred 5 out of 77 observations, the calculated uptake rates were excluded from data analysis. Because the tracer enrichment exceeded the recommended 10%, indicating an over-enrichment of these compounds in natural environmental samples, these measurements more accurately reflect potential rather than *in situ* uptake rates. NH_4_^+^ uptake rates were not corrected for “isotope dilution” (Gilbert et al., 1982; Kanda et al., 1987) and may therefore be underestimated. However, the errors would probably be small because of the short incubation times (Harrison and Harris, 1986).

For each target depth, N uptake rates and standard deviations were calculated from triplicate incubations. The average detection limit was 0.01 ± 0.01 nmol N L^−1^ h^−1^, but detection limits were calculated individually for each experiment, because calculated N uptake rates are highly dependent on the incubation time and [PN]. The ^15^N isotopic composition of the dissolved N pools were not measured due to the lack of reliable methods for all but NO_3_^−^ (see Fawcett et al., 2011). We used δ ^15^N of 6‰ when ambient NO_3_^−^ concentrations were <0.5 μmol L^−1^, and 2.5‰ when ambient NO_3_^−^ concentrations exceeded 0.5 μmol L^−1^ (Fawcett et al., 2011). For the rest of the N species, the ^15^N isotopic composition of the source pool was estimated as the natural abundance of ^15^N in atmospheric N or 0‰ (Lipschultz, 2001).

The specific uptake rate of nitrogen was calculated using Eq. 2, which is independent of [PN], or can alternatively be expressed by dividing [PN] using Eq. 1.


(2) $$\begin{array}{c} N\;15\;\mathrm{specific uptake rate}=\frac{{\left(\mathrm{atom}\%\;\mathrm{PN}\right)}_{\mathrm{final}}-{\left(\mathrm{atom}\%\;\mathrm{PN}\right)}_{\mathrm{initial}}}{{\left(\mathrm{atom}\%\;N\;\mathrm{source pool}-\mathrm{atom}\%\;\mathrm{PN}\right)}_{\mathrm{inital}}}\\ \times \frac{1\;}{\mathrm{time}} \end{array}$$

### 2.6 Phytoplankton pigment and community structure determination

To collect phytoplankton pigment samples, 1–4 L of seawater from each of the same three target depths (mentioned above) were filtered onto 25 mm GF/F filters in duplicate under a low vacuum pressure (<5–7 mm Hg). The filters were stored in cryovials and immediately frozen and stored in liquid nitrogen on board and in freezers at -80°C once ashore. Phytoplankton pigments were extracted with 3 mL of 90% acetone for 2 h in the dark at 2–8°C and measured by HPLC in the Ocean Ecology Laboratory at NASA Goddard Space Flight Center, Maryland (Hooker et al., 2012).

The relative contributions of taxa to the total Chl *a* (TChl *a*, the sum of Chl *a* and DV-Chl *a*) were calculated using the CHEMTAX program (Mackey et al., 1996). Thirteen diagnostic pigments (Supplementary Table S1) were used to associate the fractions of the TChl *a* pool with nine phytoplankton groups: diatoms (Diat), dinoflagellates (Dino), haptophytes (Type 8; Hapt_8), haptophytes (Type 6; Hapt_6), chlorophytes (Chlo), cryptophytes (Cryp), *Prochlorococcus* (Proc), *Synechococcus* (Syne), and prasinophytes (Pras). Chl *a* concentrations of pico-eukaryotes using CHEMTAX are the sum of haptophytes (Type 8), haptophytes (Type 6), chlorophytes, prasinophytes and cryptophytes. The HPLC pigment-based phytoplankton community composition determined in this study is reliable at the class level. Although this study did not combine measurements of phytoplankton taxonomic abundance using quantitative cell imaging, flow cytometry, or microscopy, at the class level, there are strong positive correlations across these different methods, except for dinoflagellates (e.g., Kramer et al., 2024).

### 2.7 Data analysis

Water masses were identified (see section 3.1) based on temperature and salinity characteristics, as well as satellite-measured sea surface temperature (SST) and sea surface height (SSH) made from August 4–11, 2016. Moderate Resolution Imaging Spectroradiometer (MODIS) Aqua level-3 SST data (8-day mean) with a horizontal resolution of 4 km was obtained from https://oceancolor.gsfc.nasa.gov/l3/. The gridded level-4 altimeter SSH data with a horizontal resolution of 0.25° × 0.25° were obtained from the Archiving, Validation and Interpretation of Satellite Oceanographic (AVISO^+^) data portal.^1^ The SSH contour of 0.2 m in the study area was used to outline the Gulf Stream edge (Muglia et al., 2022). The SSH data were also superimposed on the MODIS SST and bathymetry images to highlight the Gulf Stream path.

Chl *a* concentrations were estimated from CTD fluorescence (FL) using a linear regression (*R*^2^ = 0.94, Eq. 3) between measured fluorescence and total Chl *a* concentrations measured by HPLC.


(3) Chla=0.55×FL−0.19

The *P*^∗^ parameter was calculated following Eq. 4:


(4) $$P^{*}=\left[{\mathrm{PO}}_{4}\right]-\frac{\left[{\mathrm{NO}}_{3}^{-}\right]}{16}$$

All statistical analyses and data visualization in our study were performed using MATLAB (R2023a, MathWorks). Statistical differences between the total (and specific) N uptake rates measured at the three depths (surface, above the Chl maximum, and at the Chl maximum) were conducted via a non-parametric test (Mann–Whitney *U* test), due to the non-normal distribution of the data. Statistical differences between the total (and specific) N uptake rates among the different oceanic regimes (e.g., MAB, SAB, Slope Sea, and Gulf Stream) were also conducted via the Mann–Whitney *U* test. In addition, a redundancy analysis was conducted to link the observed N uptake rates with the phytoplankton community and environmental variables such as temperature, salinity, and concentrations of nutrients, Chl, and PN. Within the redundancy analysis, each phytoplankton species was expressed as the percentage of the total phytoplankton community.

## 3 Results

### 3.1 Study region and hydrography

**Figure 2:**
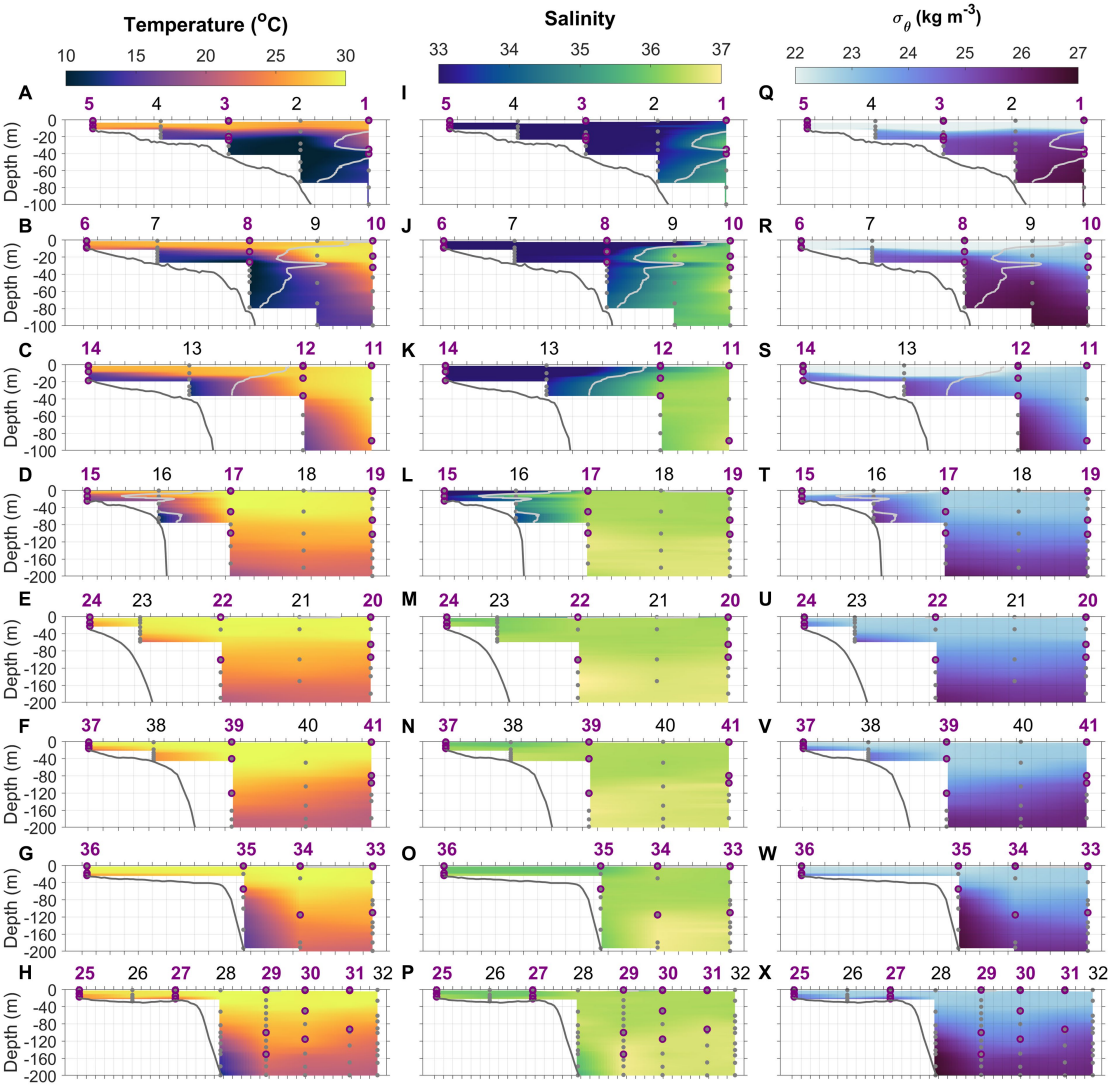
Temperature **(A–H)**, salinity **(I–P)**, and density anomaly **(Q–X)** distributions along the eight cross-shelf transects 1–8. Light gray lines in transects 1–4 denote isohaline at 34.5 which delineate the MAB and Slope Sea waters. Gray dots indicate the depths where water samples were collected using Niskin bottles. Purple circles represent the depths at which N uptake incubations were conducted. Station numbers (1–41) are indicated at the top of each panel.

The Gulf Stream, characterized by high temperature and salinity and extremely low nutrient content, is the most prominent feature of the western boundary of the North Atlantic coast (Figures 1A,B). South of Cape Hatteras, the Gulf Stream moves north, along the coast, hugging the coastline as a boundary-trapped current. Near Cape Hatteras, where the shelf-break is close to shore and the slope steepens, it separates from the coast and becomes a free jet moving north/northeast. North of Cape Hatteras, the Slope Sea, a narrow band of ocean between the Gulf Stream and the MAB continental shelf waters, forms a frontal boundary at the shelfbreak with the south-flowing subsurface Cold Pool. This Cold Pool constitutes a cold, well-mixed water mass, capped by warmer waters resulting from summertime heating inshore of the shelfbreak front (Figure 2).

**Figure 3:**
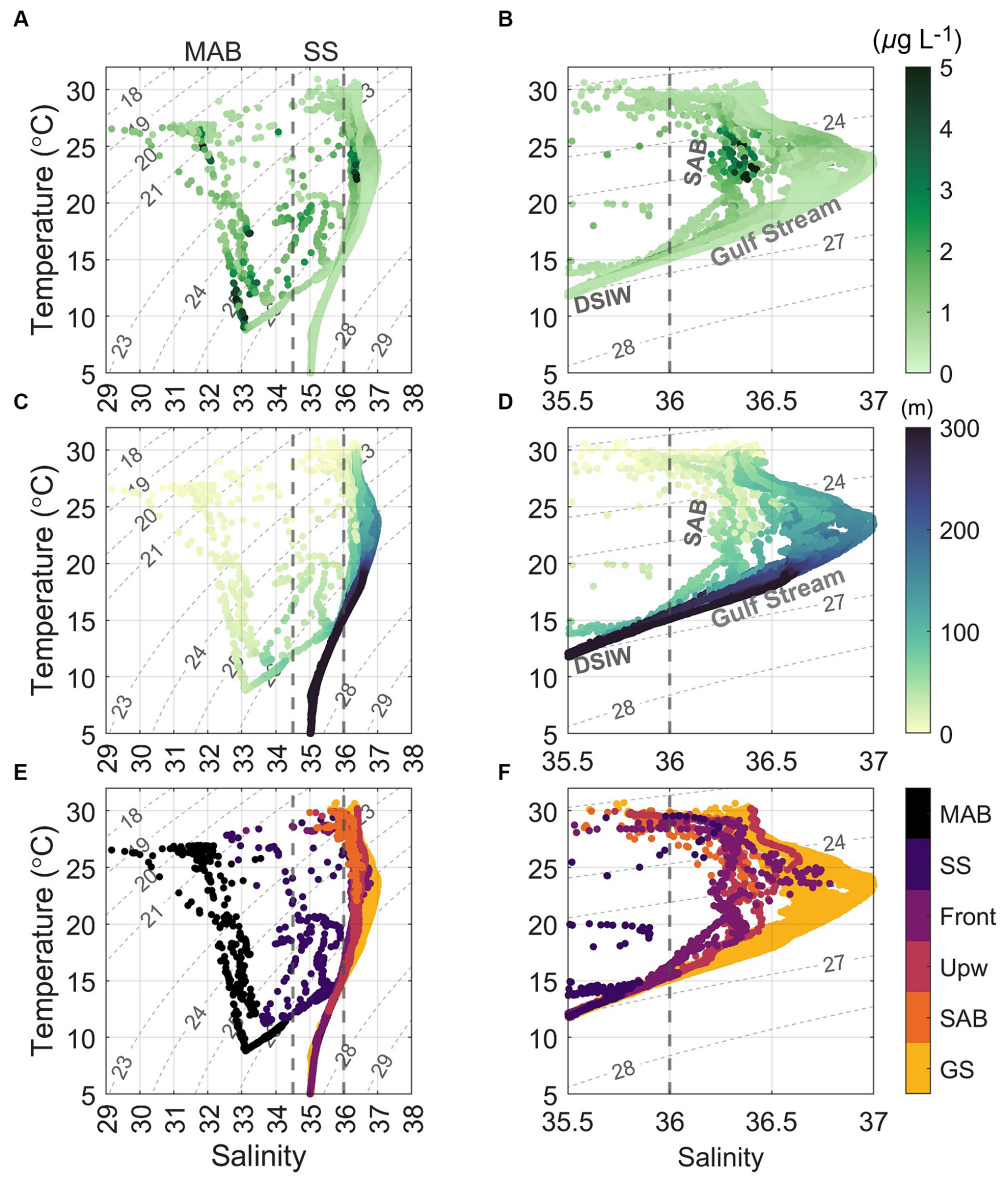
Temperature versus salinity (T − S) diagrams, superimposed with **(A,B)** chlorophyll *a* (Chl *a*) concentrations, **(C,D)** depth, and **(E,F)** color coded water masses, based on the 1 db binned CTD data collected during the August 2016 cruise. Gray contours represent isohalines for density anomaly (sigma, kg m^−3^). The right panels are the same as those on the left but are zoomed-in to focus on the salinity range from 35.5 and 37. GS, SAB, Upw, Front, SS, and MAB indicate waters from the Gulf Stream, South Atlantic Bight, upwelling, Slope Sea–Gulf Stream front, Slope Sea, and Mid-Atlantic Bight, respectively. DSIW is deep slope water (Flagg et al., 2002). The major water masses in the sampling area include MAB shelf water (S < 34.5) and Slope Sea water (34.5 < S < 36), Slope Sea-Gulf Stream frontal water (36 < S < 36.5), SAB shelf water (36 < S < 36.5), and Gulf Stream (S > 36.5). Note that in panels **(E,F)**, color coded water masses at the surface did not strictly follow the above salinity criteria.

**Figure 4:**
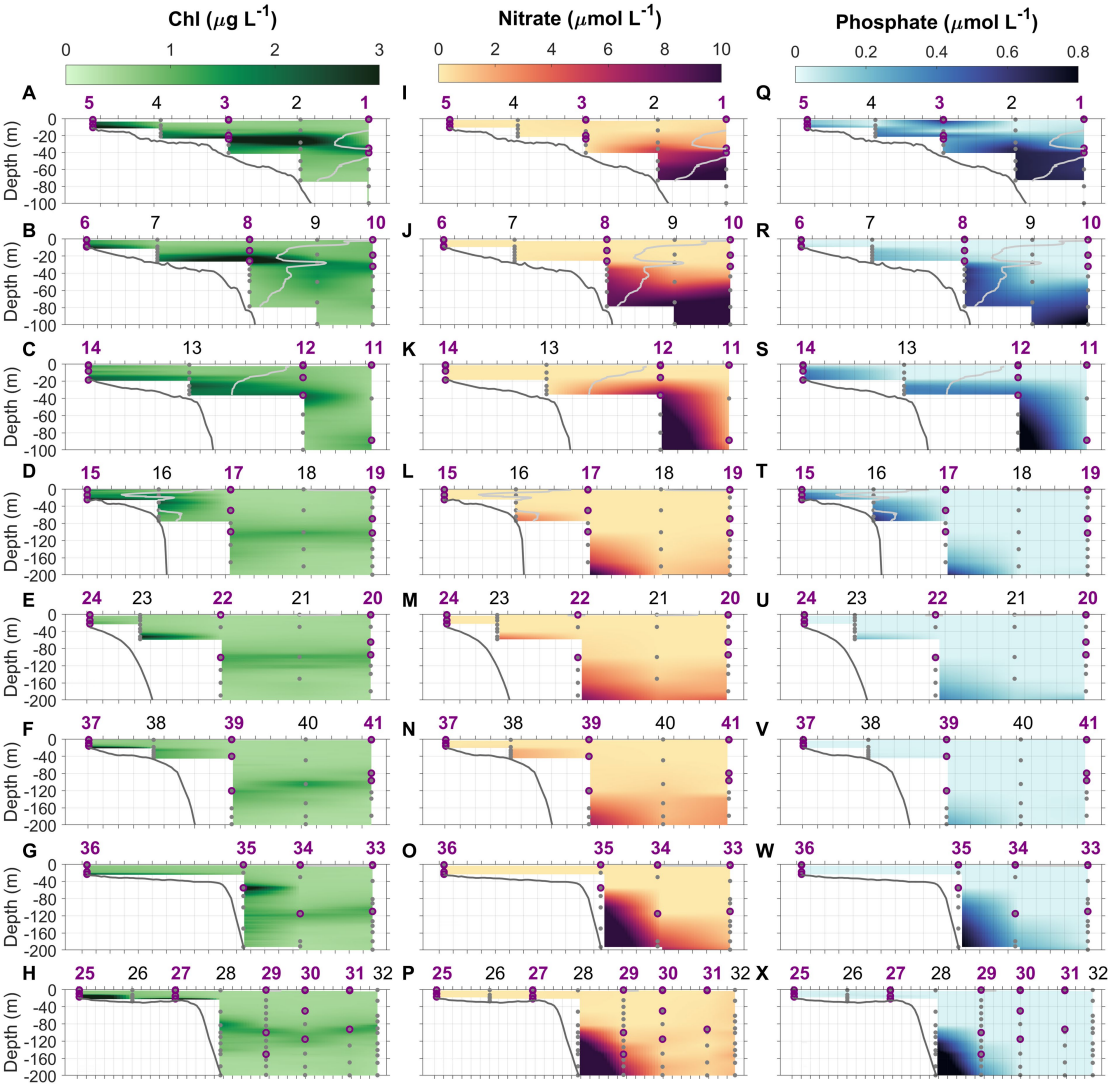
Chlorophyll (Chl, **A–H**), nitrate (NO_3_^−^, **I–P**), and phosphate (PO_4_, **Q–X**) concentrations along the eight cross-shelf transects 1–8. Light gray lines in transects 1–4 denote the 34.5 isohaline which delineates the MAB water and Slope Sea waters in transects north of Cape Hatteras. Gray dots indicate the depths where water samples were collected using Niskin bottles. Purple circles represent the depths at which N uptake experiments were conducted. Station numbers (1–41) are indicated at the top of each panel.

**Figure 5:**
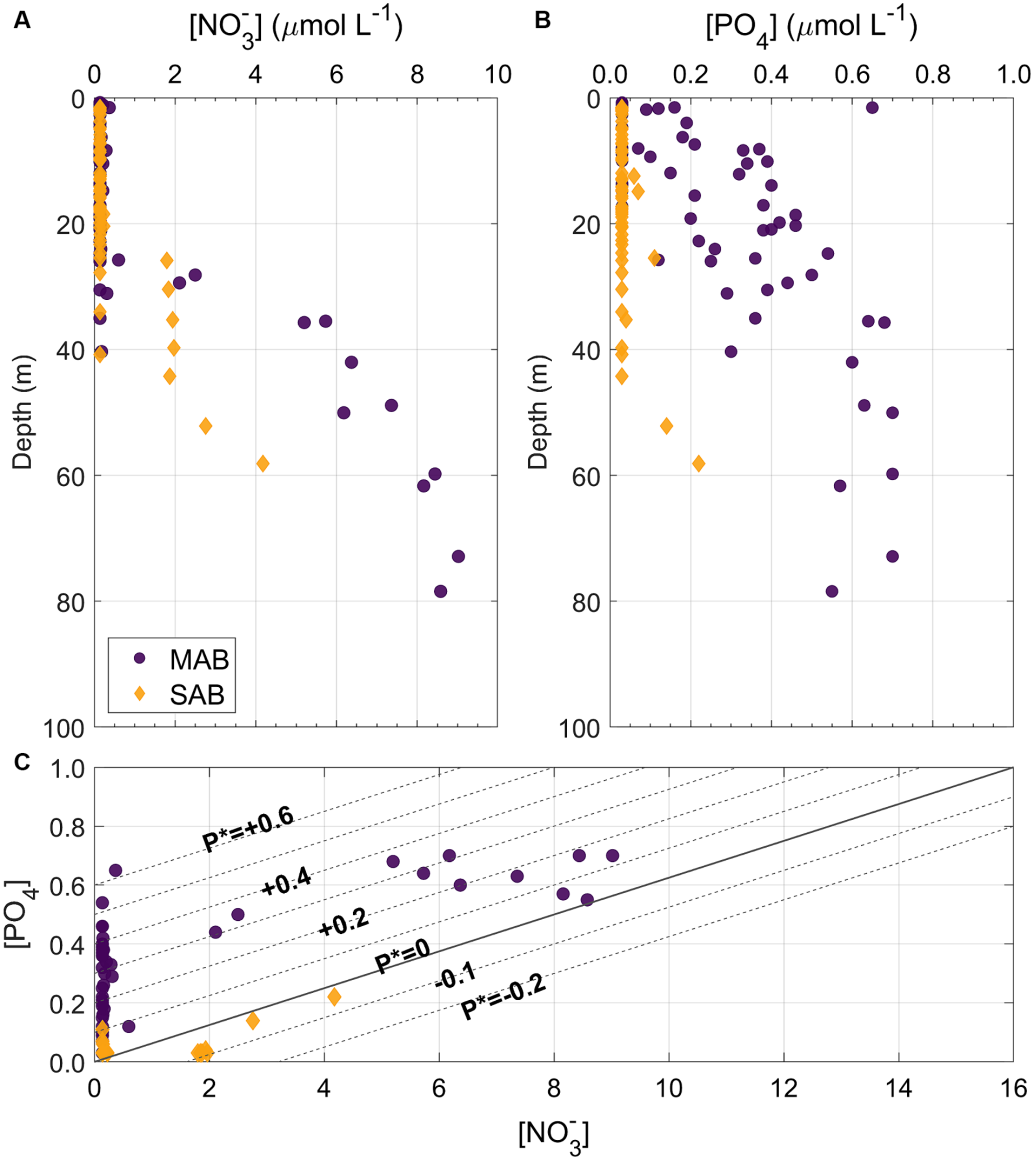
Depth profiles of **(A)** nitrate ([NO_3_^−^]) and **(B)** PO_4_ ([PO_4_]) concentrations from the MAB (dark purple circles) and SAB (orange diamonds) as well as **(C)** property vs. property plot of [NO_3_^−^]and [PO_4_], superimposed with P* contours spanning from −0.2 to +0.6 incremented by 0.1, where P* = [PO_4_] – [NO_3_^−^]/16.

**Figure 6:**
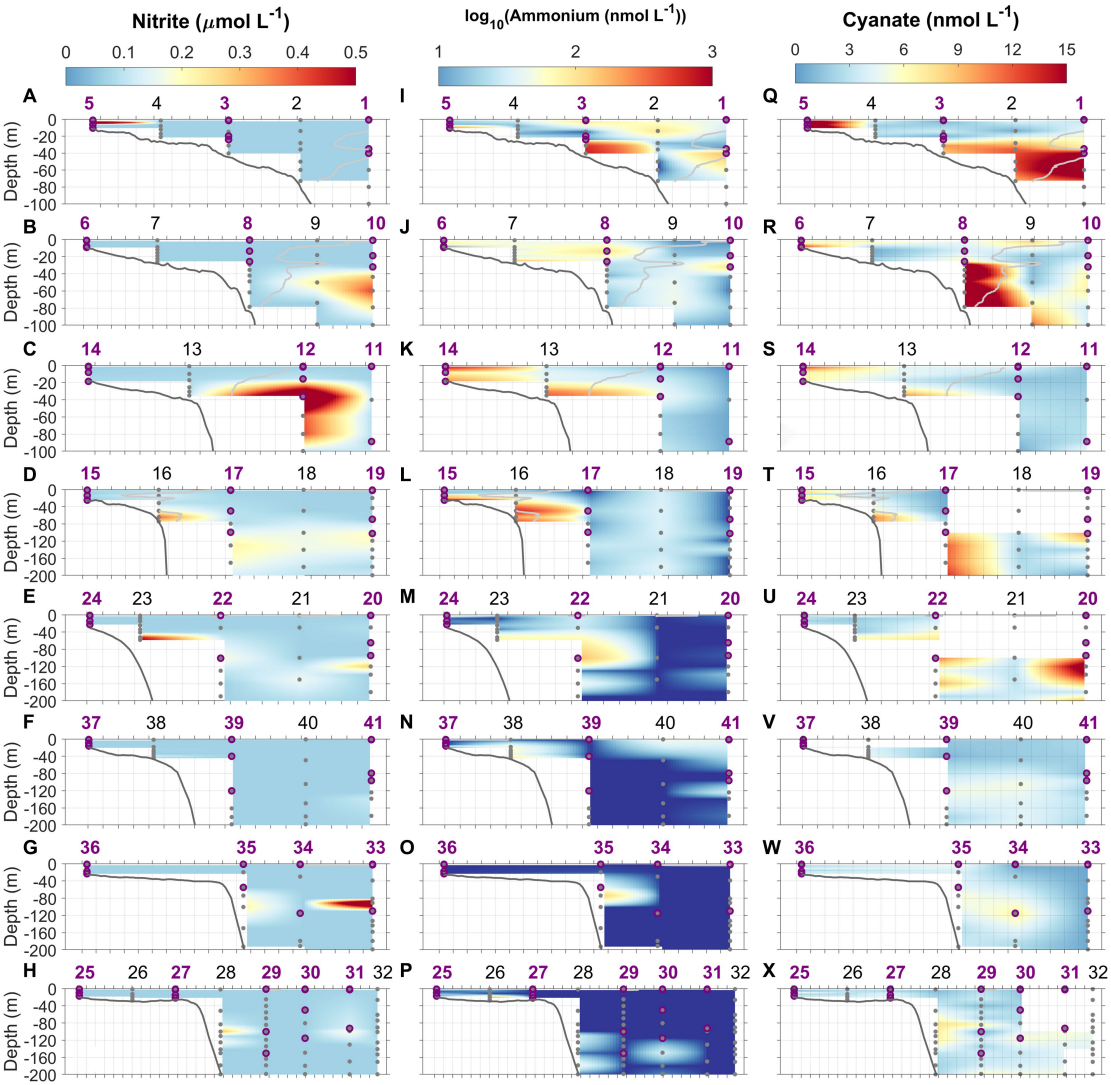
Nitrite (NO_2_^−^, **A–H**), ammonium (NH_4_^+^, log transformed, **I–P**), and cyanate **(Q–X)** concentration distributions along the eight cross-shelf transects 1–8. Light gray lines in transects 1–4 denote isohaline at 34.5 which delimit the MAB water and Slope Sea. Gray dots indicate the depths where water samples were collected using Niskin bottles. Purple circles represent the depths at which N uptake incubations were conducted. Station numbers (1–41) are indicated at the top of each panel.

Our study area was at the intersection of the southwest flowing MAB shelf water Cold Pool and shelfbreak jet, and the north flowing SAB shelf water and Gulf Stream, thus featuring very distinctive physical (Seim et al., 2022; Figures 2, 3) and biogeochemical properties (Figures 4–6). The contrasts between the four distinct oceanic regimes were identified through sea surface temperature (SST) and sea surface height (SSH) maps (Figure 1), sections of temperature, salinity, and density anomalies along the 8 cross-shelf transects (Figure 2), temperature (T) − salinity (S) diagrams (Figure 3), and sections of chlorophyll and inorganic nutrient concentrations (Figure 4). North of Cape Hatteras, the MAB shelf and Slope Sea waters were significantly cooler (Figures 2A–D). At some MAB nearshore stations, low-salinity waters (S < 32) were observed, likely due to the introduction of low-salinity estuarine waters from the Chesapeake Bay (Flagg et al., 2002). The MAB shelfbreak frontal system (shelf − slope) had a more pronounced temperature and salinity gradient due to interactions with the Cold Pool waters intruding from the north (Figures 2A–D,I). The MAB shelf and Slope Sea water were distinguished based on temperature and salinity characteristics (Figure 3; Todd, 2020). Based on the observed gradients we operationally grouped stations into MAB shelf water, when salinity was less than 34.5, and Slope Sea water, when salinity was between 34.5 and 36 (Figure 3).

In contrast, SAB shelf waters south of Cape Hatteras in August were difficult to distinguish from Gulf Stream waters based on temperature and salinity alone (Figure 3; Seim et al., 2022), due to the strong influences from the adjacent Gulf Stream, limited riverine discharge, and seasonal warming. Therefore, there wasn’t a clearly defined frontal zone where SAB shelf waters and the Gulf Stream water interacted (Figures 1B, 2E–H,M–P,U–X). Thus, the SAB shelf water was considered to be waters extending from the coastline to the 200 m isobath. Notably, upwelling occurred at the SAB shelfbreak, including Stations 23, 28, and 35, that was accompanied by lower subsurface water temperatures (Figures 2E,G,H), isopycnal uplifting at *σ* = 24.5 kg m^−3^ (Figures 2U,W,X), and higher nutrient concentrations (Figures 4M,O,P,U,W,X).

### 3.2 Chl and nutrient distributions

At inner shelf stations where water depths were <30 m, Chl maxima were observed near the bottom, while at outer shelf and offshore oceanic stations, subsurface Chl maxima were prominent features (Figures 4A–H). The depth of the subsurface Chl maximum deepened from shelf to offshore, it was 21 ± 7 m in the outer shelf of the MAB, 33 ± 4 m in the Slope Sea, and 102 ± 11 m in the Gulf Stream (Figures 4A–H; also see Figure 3 in Selden et al., 2021). At shelfbreak stations south of Cape Hatteras where upwelling occurred, e.g., Stations 23, 28, and 35, subsurface Chl maxima were deeper, appearing at 52, 82, and 54 m, respectively (Figures 4E,G,H; Supplementary Figures S2A,G,H). In addition, the Chl concentrations within subsurface Chl maxima were highly variable among different water masses—higher in both the MAB and SAB shelf water and extremely low within the Gulf Stream (Figures 3A, 4A–H).

Distributions of NO_3_^−^ and PO_4_ (sum of HPO_4_^2−^ and PO_4_^3−^) concentrations were described in Selden et al. (2021). Briefly, NO_3_^−^ was mostly depleted throughout both the MAB and SAB during the period of the survey, however, high PO_4_ concentrations, relative to NO_3_^−^ and the Redfield ratio, were observed in shelf waters in the MAB, unlike the SAB where both NO_3_^−^ and PO_4_ concentrations were depleted (Figures 4I–L,Q–T, 5). At Chl hotspots (>2 μg Chl L^−1^) in the MAB, e.g., at Stations 2 and 8 (Figures 4A,B), the high Chl concentrations appeared to be located at depths at the top of the nitra- and phospho-clines (Figures 4I,J; Supplementary Figures S1B,C). At Stations 23, 28, and 35, higher subsurface NO_3_^−^ and PO_4_ concentrations were also observed (Figures 4M,O,P,U,W,X; Supplementary Figures S2A,G,H), as a result of upwelling of deeper nutrient-rich water. In the Gulf Stream, both NO_3_^−^ and PO_4_ concentrations were depleted in the upper 150–200 m. Supplementary Figures S1, S2 show detailed depth profiles of Chl and nutrient concentrations at representative stations.

In general, NO_2_^−^ concentrations in the study area ranged from below the detection limit (0.07 μmol L^−1^) to 0.92 μmol L^−1^ (Figures 6A−H). For most stations, NO_2_^−^ concentrations were not detectable throughout the water column, and subsurface NO_2_^−^ maxima were very ephemeral (Figures 6A–H; Supplementary Figures S1, S2). As for NO_2_^−^, NH_4_^+^ concentrations ranged from below the detection limit (10 nmol L^−1^) to 960 nmol L^−1^ (Figures 6I–P). We observed that NH_4_^+^ concentrations were often highest near the bottom (up to 0.96 μmol N L^−1^) at shallow inner-shelf stations. At offshore stations where the water depths were greater, two types of vertical NH_4_^+^ distributions were found, those with and without the presence of NH_4_^+^ maxima (e.g., Supplementary Figures S1, S2). NH_4_^+^ concentrations on the SAB shelf were much lower compared to the MAB shelf (Figures 6M–P), and NH_4_^+^ maxima were absent at some stations in the SAB. At stations in the Gulf Stream, NH_4_^+^ concentrations were mostly below the detection limit, and both the primary NO_2_^−^ and NH_4_^+^ maxima were absent or barely detectable (Figure 6). Cyanate concentrations varied from below the detection limit (0.4 nmol L^−1^) to 25 nmol L^−1^ (Figures 6Q–X). Similar to NH_4_^+^, higher cyanate concentrations were observed in the bottom waters of the MAB shelf (e.g., Stations 6 and 13, and Supplementary Figures S1A,G) suggestive of a sedimentary source. Like NH_4_^+^, cyanate maxima were ephemeral, occurring in the subsurface waters at some stations (e.g., Stations 1, 2, 19, 20, and 34) but absent at others (e.g., Stations 12, 25, 35, and 41). Supplementary Figures S1, S2 show typical profiles of cyanate. Urea concentrations were below the detection limit (0.08 μmol L^−1^) for all samples. Total dissolved primary amines concentrations ranged 0.05–0.4 μmol N L^−1^, with the mean concentration of 0.17 ± 0.08 μmol N L^−1^ at depths above and at the depth of Chl maximum (data not shown).

### 3.3 Absolute and specific N uptake rates in the euphotic zone

**Figure 7:**
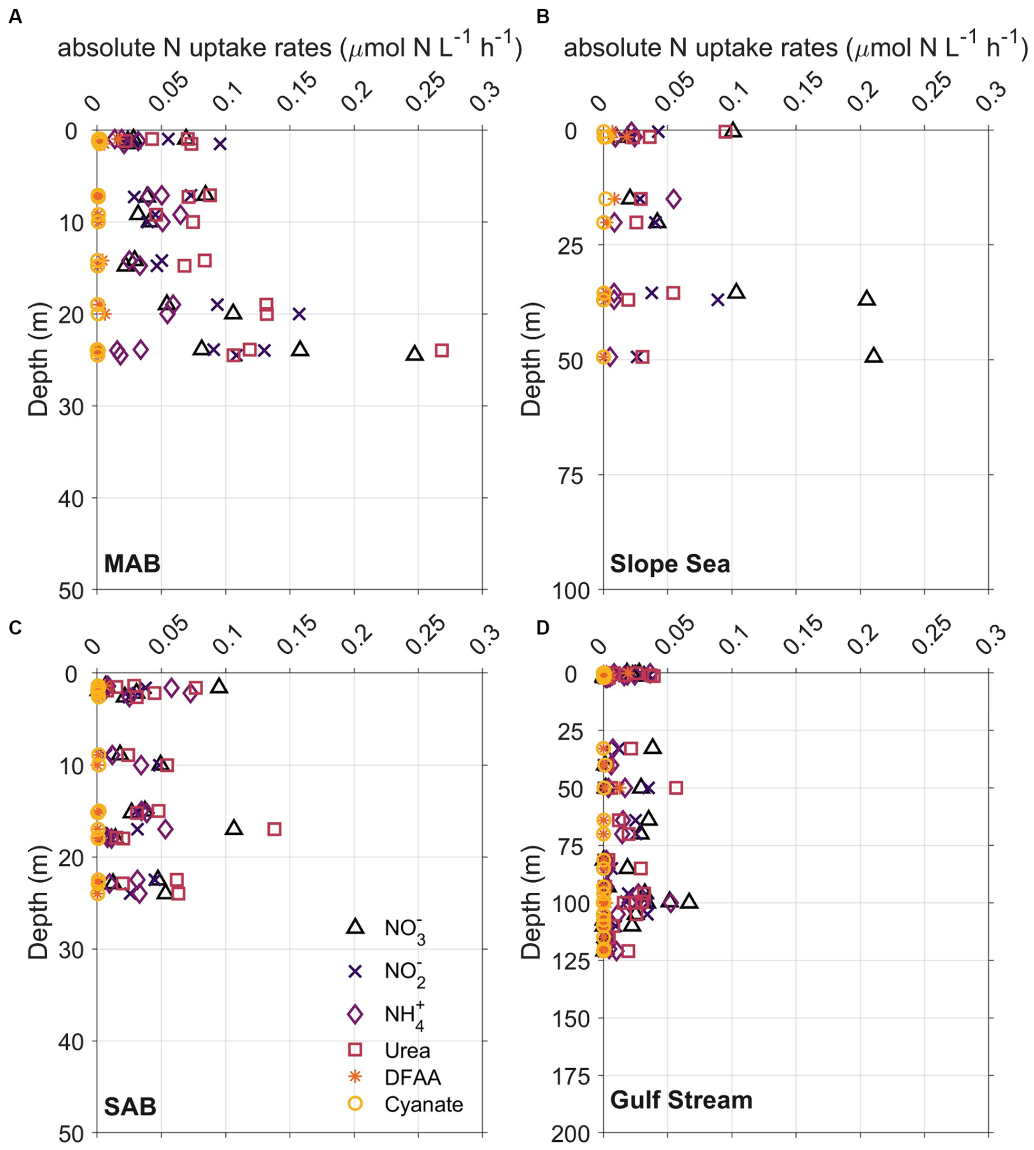
Vertical distribution of whole community N uptake rates including nitrate (NO_3_^−^, triangles), nitrite (NO_2_^−^, crosses), ammonium (NH_4_^+^, diamonds), urea (squares), dissolved free amino acids (DFAA, asterisks), and cyanate (circles) in **(A)** Mid-Atlantic Bight (MAB), **(B)** Slope Sea, **(C)** South Atlantic Bight (SAB), and **(D)** Gulf Stream waters. The depth range of *y* axis at each panel is different because depths of Chl maximum deepened from onshore to offshore.

**Figure 8:**
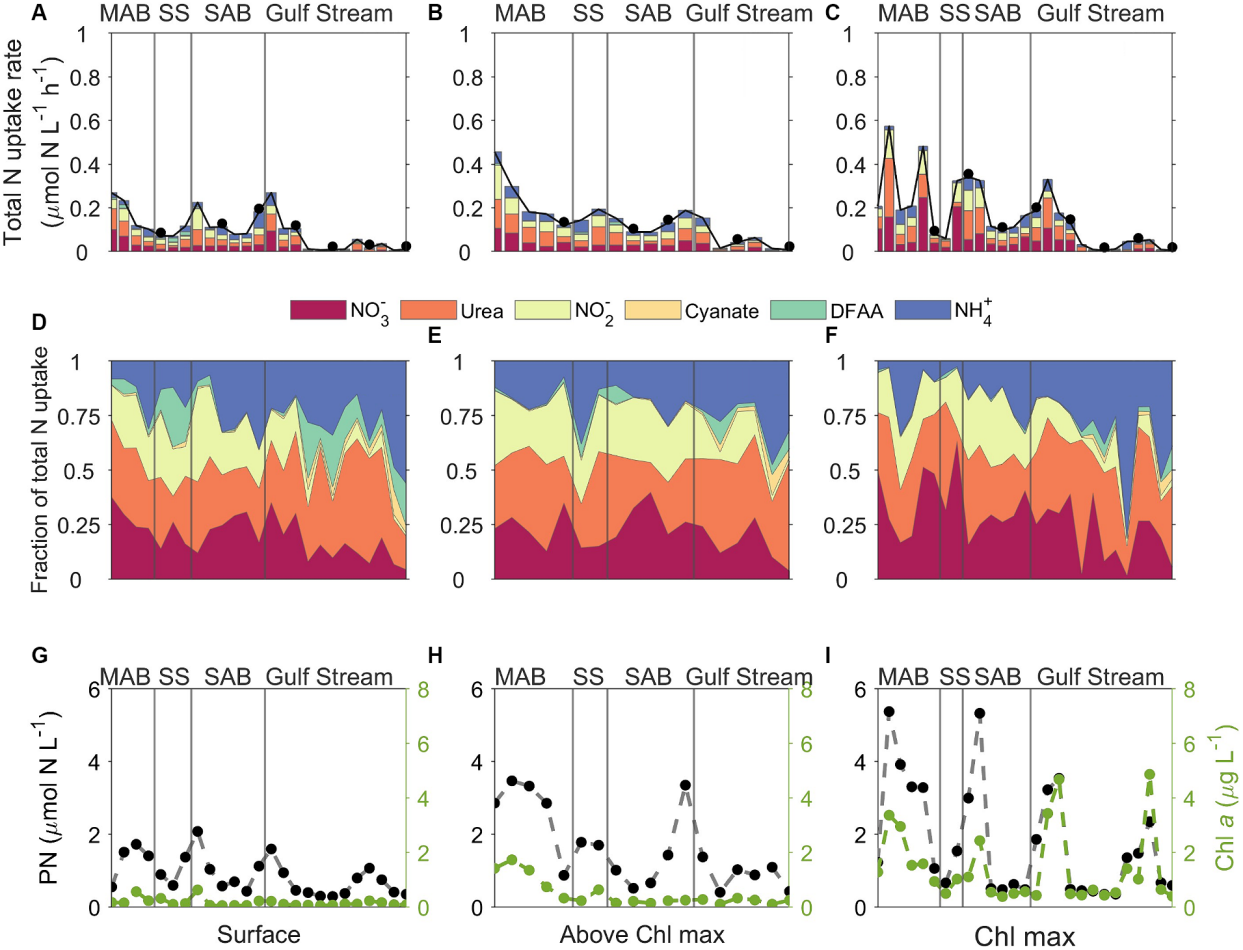
Plots of **(A–C)** stacked N uptake rates, **(D–F)** the fraction of total N uptake contributed by the various N species measured, and **(G–I)** corresponding particulate nitrogen (PN, black dots) and Chl *a* (green dots) concentrations across different regions which delimited by gray lines. MAB, SS, SAB stand for Mid Atlantic Bight, Slope Sea, and South Atlantic Bight, respectively. The left, middle, and right panels represent data from surface, above the Chl maximum, and Chl maximum depths, respectively. The upper two panels share the same color palette, and each color represents one of the six tested N species. DFAA in the color legend stands for dissolved free amino acids. Black dots in the upper panel indicate that incubation was conducted at nighttime.

Although the vertical distribution of N uptake rates appeared to be increasing with depth at certain stations and for some N compounds (Figures 7, 8A–C), total absolute N uptake rates in surface waters, above the Chl maximum, and at the Chl maximum were not significantly different (*p* > 0.05) from each other. Nighttime uptake rates were comparable to those daytime uptake rates observed at the neighboring stations within the same sampling region (Figures 8A–C). Among the six N species examined, urea uptake rates were the highest, accounting for ~27% of the total measured N uptake rates. NO_3_^−^ and NH_4_^+^ uptake rates were comparable across the study region, and together made up ~40% of the total measured N uptake rates (Figures 7, 8D–F). Amino acids and NO_2_^−^ uptake rates were similar to each other, together representing ~30% of the total measured N uptake (Figures 7, 8D–F). Cyanate-N uptake rates were very low, accounting for just 1.2% of the total N uptake on average (Figures 7, 8D–F).

**Figure 9:**
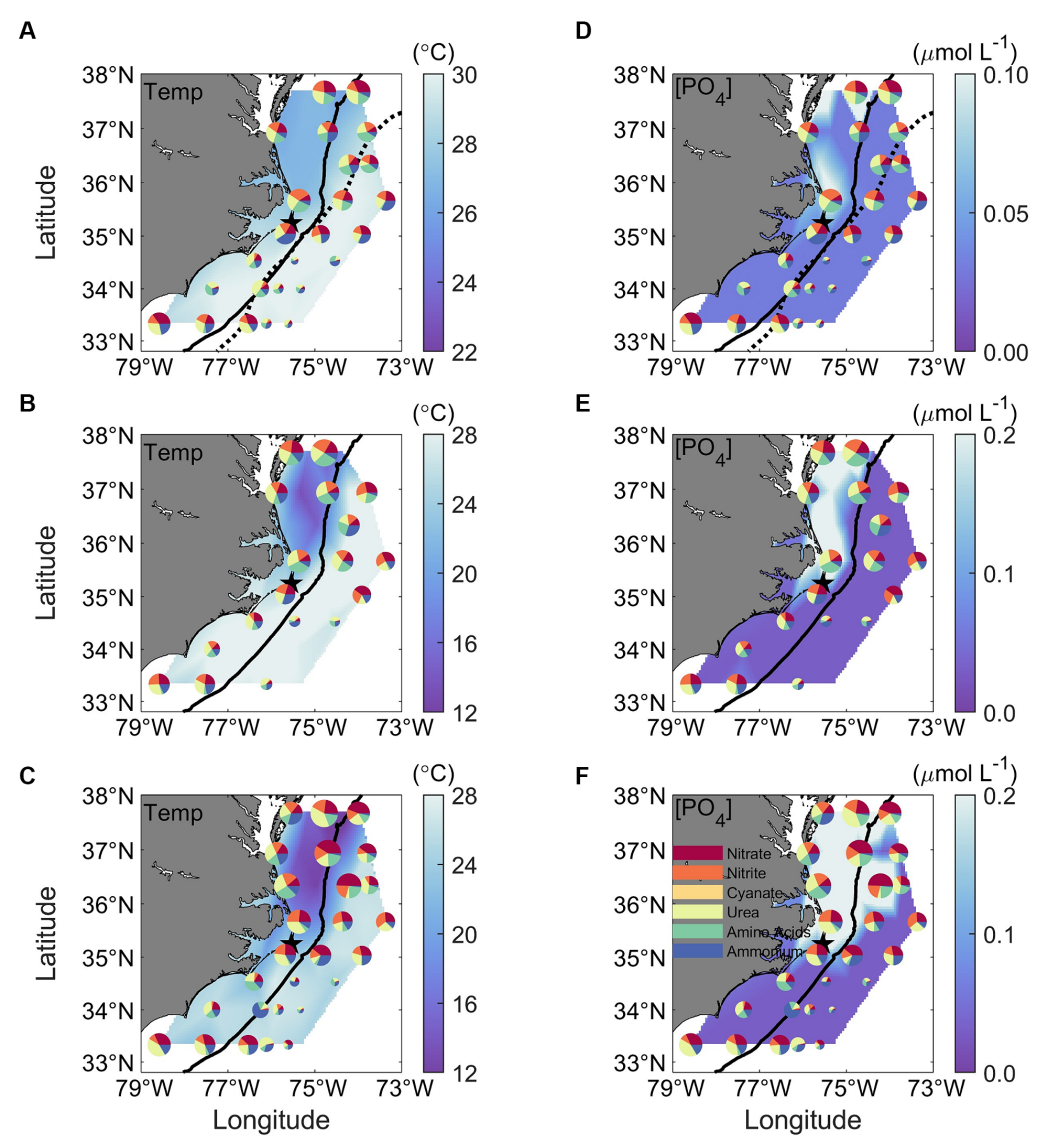
Spatial distribution of N uptake rates contributed by the six different N species, superimposed with **(A–C)** *in-situ* temperature and **(D–F)** PO_4_ concentrations ([PO_4_]) measured at the surface (upper panels), above the Chl maximum (middle panels), and at the Chl maximum depth (bottom panels). The size of each pie in the figure represents the magnitude of the total N uptake, and each colored slice corresponds to one of the six tested N species. The color legend is provided in panel **(F)**. Contrasts in temperature and PO_4_ concentrations among the MAB, SAB, SS, and GS regions are observed. The thick black solid line in all panels mark the 200-m isobaths. The black dotted lines in upper panels represent the Gulf Stream Edge.

There were remarkable spatial differences in the magnitude of absolute N uptake rates across the different regimes sampled (Figures 8, 9). Total absolute N uptake rates ranged from 0.13–0.69 μmol N L^−1^ h^−1^ in the MAB (Figures 8A–C), with combined uptake rates of urea, amino acids, and NO_3_^−^ representing ~70% of the total measured N uptake, and uptake of cyanate accounting for only ~0.4% of the total measured N uptake rates (Figures 8D–F). In the Slope Sea, total measured N uptake rates (mean = 0.23 μmol N L^−1^ h^−1^, *n* = 8) were comparable to those observed in the MAB, ranging from 0.11–0.42 μmol N L^−1^ h^−1^ (Figures 8A–C). Like the MAB, about 73% of the total observed N uptake in the Slope Sea was from urea, amino acids, and NO_3_^−^ (Figures 8D–F). In the SAB, total N uptake rates were lower, ranging from 0.03 to 0.33 μmol N L^−1^ h^−1^ (Figures 8A–C). Together, NO_3_^−^, urea, and NH_4_^+^ uptake comprised ~73% of the total N uptake there (Figures 8D–F). Total measured N uptake rates were significantly greater (*p* < 0.05, Mann–Whitney U test) in the MAB region (mean = 0.34 μmol N L^−1^ h^−1^, *n* = 15) than the SAB region (mean = 0.13 μmol N L^−1^ h^−1^, *n* = 17). In the Gulf Stream, total measured N uptake rates were the lowest, ranging from 0.004–0.18 μmol N L^−1^ h^−1^ (mean = 0.062 μmol N L^−1^ h^−1^, *n* = 32; Figures 8A–C, 9). Cyanate uptake rates within the Gulf Stream were greater than those observed in other regions and represented up to ~11% of the total measured N uptake rates at some stations (Figures 8D–F).

**Figure 10:**
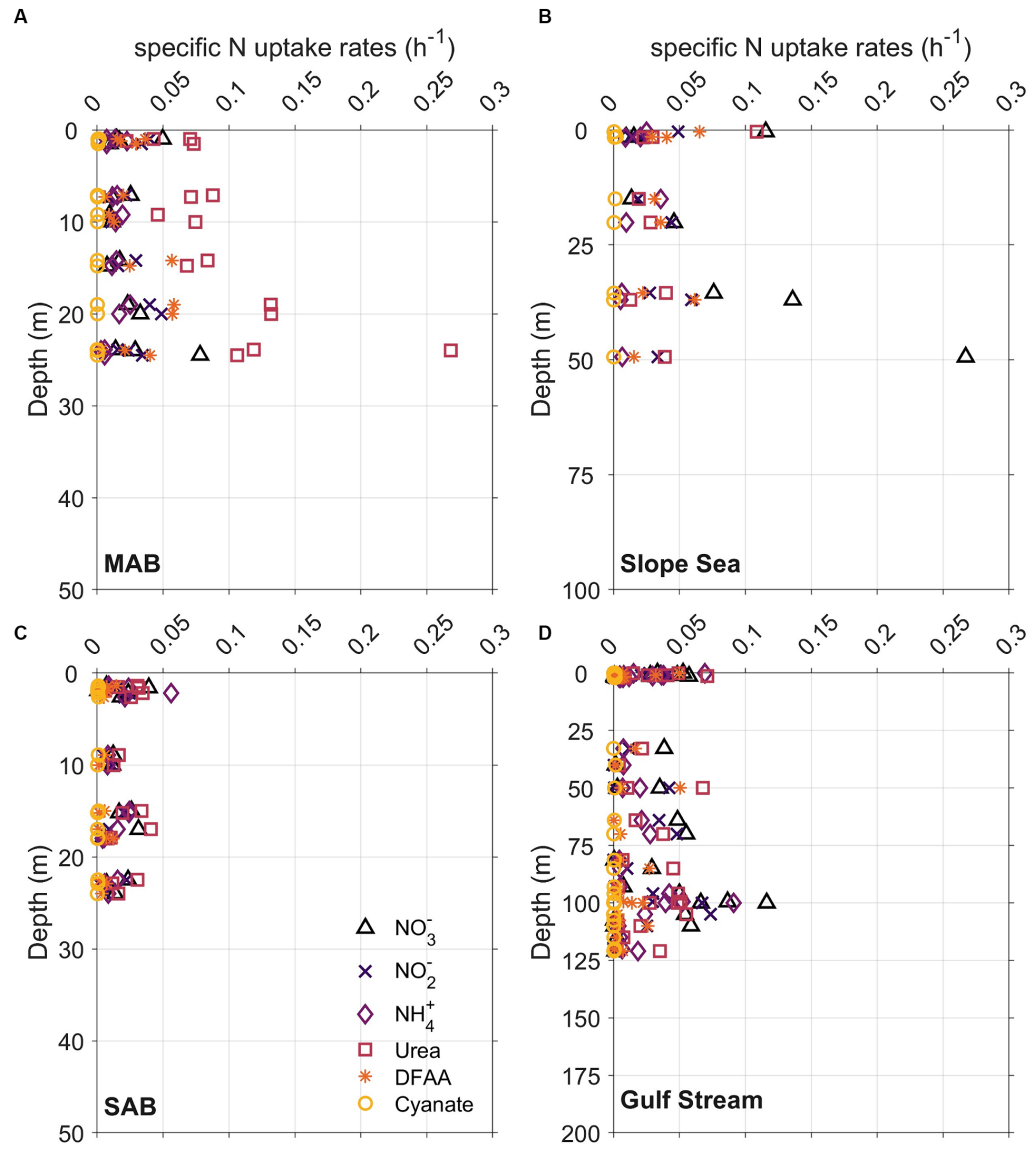
Same as Figure 7, but for specific N uptake rates, which are independent from particulate nitrogen concentrations.

Specific N uptake rates (h^−1^) that are independent from PN showed a similar trend as the absolute N uptake rates (μmol N L^−1^ h^−1^) (Figure 10; Supplementary Figures S3A–C). The average specific N uptake rates were 0.031, 0.026, 0.020, 0.017, 0.016, and 0.0005 h^−1^ for NO_3_^−^, urea, NO_2_^−^, NH_4_^+^, and amino acids, and cyanate, respectively. Similar to the absolute N uptake rates, the specific uptake rates of urea, NO_3_^−^, and NH_4_^+^ were higher than those for NO_2_^−^, amino acids, and cyanate (Supplementary Figures S3D–F). In vertical profiles, total specific uptake rates did not exhibit significant differences across the three depths examined. Spatially, total specific uptake rates were lowest in the SAB and Gulf Stream (Figure 10; Supplementary Figure S4) and significantly higher in the MAB (*p* < 0.05, Mann–Whitney *U* test).

### 3.4 Phytoplankton community composition in the euphotic zone

**Figure 11:**
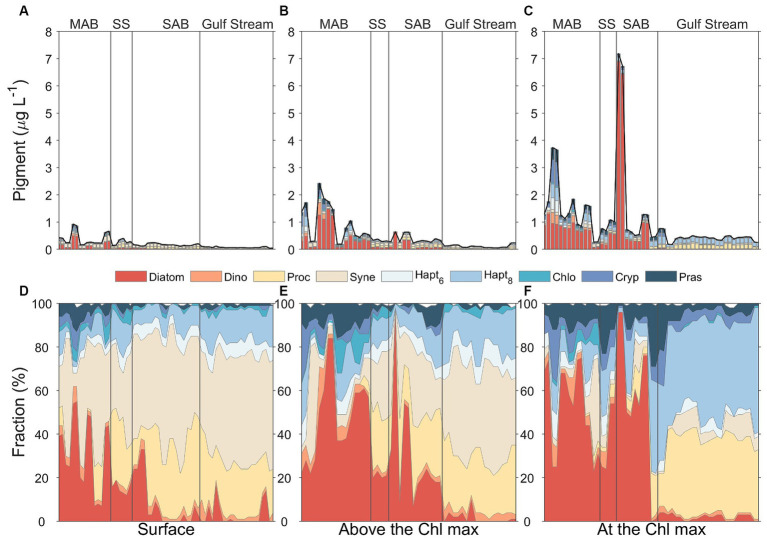
Phytoplankton community composition at the surface water (**A,D**, left panels), above the Chl maximum (**B,E**, middle panels), and Chl maximum depths (**C,F**, right panels) in four distinct regimes separated by the gray solid lines, including Mid-Atlantic Bight (MAB), Slope Sea (SS), South Atlantic Bight (SAB), and Gulf Stream. The total stacked bar length in upper panels represent the total Chl *a* concentrations, and the composition graph in lower panels reflects the relative contributions of different phytoplankton groups to total Chl *a* determined from CHEMTAX assessment of diagnostic pigments.

Overall, diatoms, picocyanobacteria (*Prochlorococcus* and *Synechococcus*), and haptophytes (Type 8) were the dominant groups in the study area based on CHEMTAX analysis of pigment data (Figure 11). Prasinophytes were also relatively prevalent in the surface mixed layer and at the depth of the chlorophyll maximum in the MAB, Slope Sea, and SAB (Figure 11). The phytoplankton community also displayed distinctive biogeographical patterns with diatoms dominating the phytoplankton community composition in the MAB and Slope Sea, transitioning to picocyanobacterial dominance in the Gulf Stream. Phytoplankton community composition in the SAB appeared to be vertically stratified with picocyanobacteria dominant in surface waters and diatoms becoming increasingly dominant with depth (Figure 11). The Gulf Stream community was characterized by high concentrations of picocyanobacteria in both surface and subsurface waters, however, haptophytes (Type 8) and *Prochlorococcus* co-dominated at the depth of the chlorophyll maximum. These three groups contributed 93% of the total Chl *a* in Gulf Stream.

The vertical profiles of phytoplankton biomass differed between water masses (Figure 11). Diatoms and prasinophytes showed clear subsurface maxima layers in all regions except for the Gulf Stream. For the Gulf Stream, haptophytes (Type 8), and *Prochlorococcus* showed clear subsurface maxima. *Synechococcus* was more prevalent in surface waters in all regions and its relative dominance decreased with depth. Other groups [haptophytes (Type 6), chlorophytes, and cryptophytes] also showed vertical variations, but their concentrations were relatively low throughout the water column and in all regions.

## 4 Discussion

This study marked the first instance of simultaneously measuring uptake rates for six different N compounds across contrasting regions. Considering the significant differences in sampling time, area, and associated environmental factors compared to previous studies, the reported N uptake rates here should be regarded as robust. Within the northern MAB, the N uptake rates measured in our study generally fell within the same range as previously reported for this area (e.g., Glibert et al., 1991; Filippino et al., 2011), however, the uptake rates of individual species (e.g., NH_4_^+^ and NO_3_^−^) in the MAB were much higher than those measured in Georges Bank (e.g., Harrison and Wood, 1988). Within the Gulf Stream, the measured N uptake rates (e.g., NH_4_^+^ and NO_3_^−^) in our study were significantly higher than those reported in Glibert et al. (1988), which might have been be due to the high enrichment of ^15^N in some incubation bottles. However, NH_4_^+^, NO_3_^−^ and urea uptake rates were generally comparable with those observed in other parts of the Atlantic Ocean (e.g., Rees et al., 2006; Painter et al., 2008a,b). Cyanate uptake rates represented up to ~11% of the total measured N uptake rates at some stations within the Gulf Stream, in agreement with the results from Widner and Mulholland (2017), however, the higher contribution of cyanate may simply be due to the lower uptake rates of other N species within the Gulf Stream.

### 4.1 N uptake rates with respect to ambient P concentrations

Chronic or transiently low P availability can constrain biological productivity in aquatic systems (Trommer et al., 2013; Karl, 2014; Duhamel et al., 2021; Hashihama et al., 2021). In addition, a growing body of literature demonstrates the pervasive occurrence of N − Fe or N − P co-limitation of phytoplankton growth across diverse ocean regimes (Graziano et al., 1996; Saito et al., 2014; Browning and Moore, 2023). Nutrient amendment experiments in the North Atlantic subtropical gyre (low-nutrient, low-chlorophyll) confirmed that combining P with N induces larger increases in chlorophyll and primary productivity than N additions alone, emphasizing the role of dissolved P as a secondary limiting factor on phytoplankton growth (Graziano et al., 1996; Moore et al., 2008; Sedwick et al., 2018). Although DOP might serve as alternative P source when PO_4_ is depleted, its concentrations generally decrease with increasing PO_4_ stress, as indicated by more negative P* values, yielding the lowest DOP level in regions like North Atlantic (as seen in Liang et al., 2022), thus, the SAB and Gulf Stream provinces might experience both inorganic and organic P stress (Mather et al., 2008).

**Figure 12:**
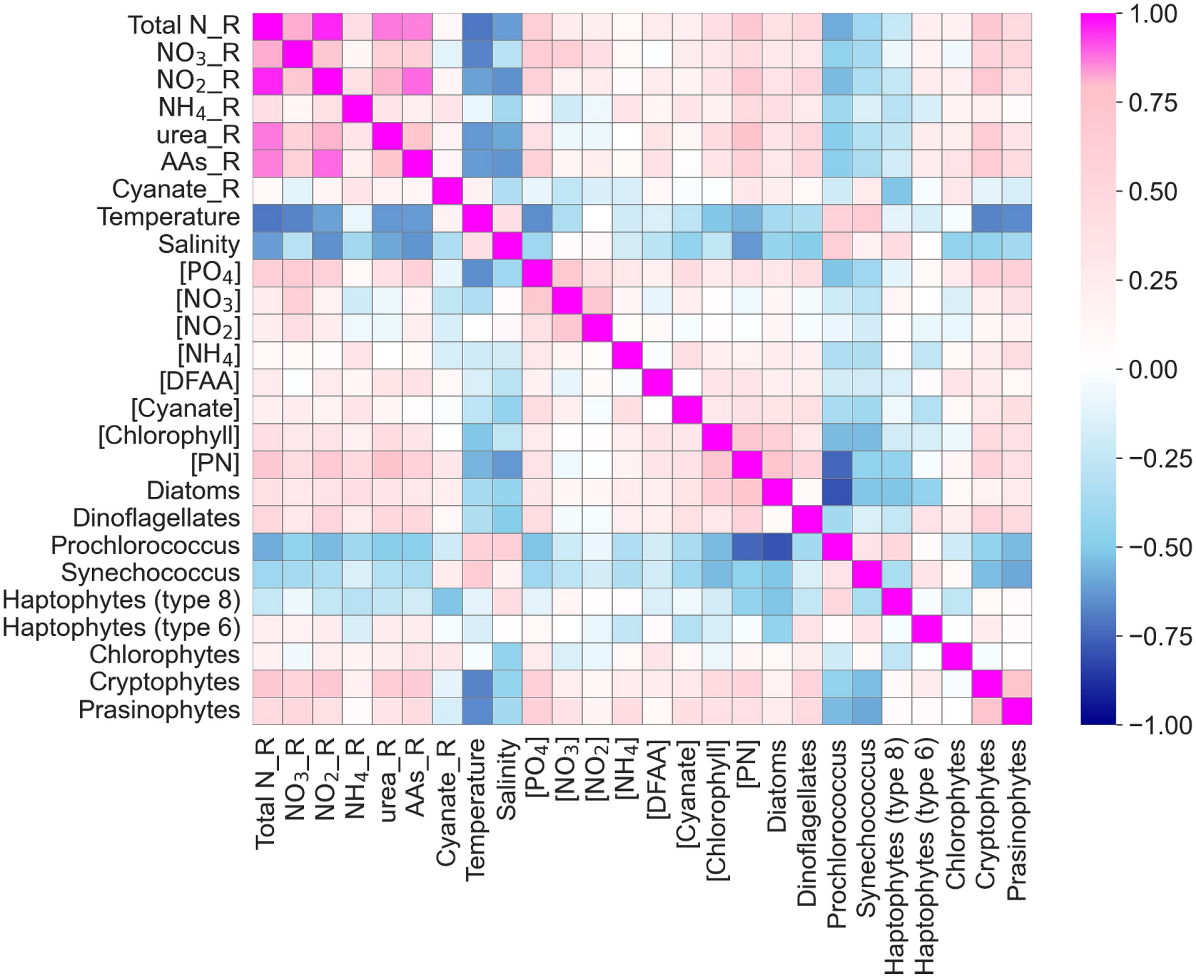
Heatmap of correlation coefficients among N uptake rates and environmental factors including temperature, salinity, ambient nutrient concentrations ([PO_4_], [NO_3_^−^], [NO_2_^−^], [NH_4_^+^], [DFAA], [Cyanate]), chlorophyll concentrations, particulate nitrogen [PN], and phytoplankton species (in %). Total N_R, NO_3__R, NO_2__R, NH_4__R, urea_R, AAs_R, Cyanate_R stands for the uptake rate of total nitrogen, nitrate, nitrite, ammonium, urea, amino acids, and cyanate, respectively. DFAA stands for dissolved free amino acids.

The Cold Pool contains a reservoir of nutrients that can sustain phytoplankton productivity on the MAB shelf during the spring and summer months (Flagg et al., 1994; Hales et al., 2009). Despite significant differences in temperature and salinity between the MAB and SAB, this study found excess PO_4_ in the MAB waters (Figures 4, 5). The positive P* values across the northern MAB suggest an excess PO_4_ relative to N and the Redfield Ratio, a trend contrasting with the mostly near-zero or negative P* values observed in the SAB (Figure 5C). This was likely due to: (1) the MAB being influenced by the Cold Pool waters intruding from the sub-Arctic whose source waters have already undertaken denitrification, resulting in an excess PO_4_ relative to NO_3_^−^ (Harrison and Li, 2007; Fennel, 2010; Sherwood et al., 2021), and (2) the SAB shelf region has more direct interactions with the oligotrophic Gulf Stream and has limited riverine inputs (Andres et al., 2023). Specific N uptake rates were significantly higher in the MAB than the SAB (*p* < 0.05), showing a positive correlation with ambient P concentrations (*r* = 0.31, *p* < 0.05, Figure 12). This result suggests a N-P synergy, such that increased P availability enhances phytoplankton’s ability to assimilate and utilize available N, as has been observed in the northern Gulf of Mexico (Turner and Rabalais, 2013); whereas when P is limited, organisms may not be able to utilize available nitrogen as efficiently, leading to lower apparent nitrogen uptake rates.

Under various growth conditions, phytoplankton employ optimal allocation strategies, allowing them to allocate N and P to specific cellular functions necessary for survival (and growth) and thereby offering them some plasticity in their N:P ratios. For example, phytoplankton N:P ratios can be higher when growing under competitive equilibrium conditions with resource limitations (e.g., N, P, and light) that requires allocating resources to acquisition assembly. Conversely, phytoplankton N:P ratios can be lower during exponential growth when cell metabolism assembly takes priority (Klausmeier et al., 2004; Armin et al., 2023). Observations suggest a nuanced pattern where fast-growing species, like diatoms, have relatively stable but P-rich elemental compositions, while slower-growing species, such as cyanobacteria, exhibit more flexible elemental stoichiometry (Hillebrand et al., 2013). In addition, at low nutrient concentrations, diffusive nutrient transport was found to increase linearly with phosphorous concentrations (Armin et al., 2023). In the context of this study, diatoms dominated both the MAB and SAB regions, hence the demand for P at their fast-growing phase might have been high. Consequently, the synergistic response, higher N uptake rates (and perhaps growth rates) in the MAB where there was excess P, may have been promoted to sustain the increased allocation to biosynthetic and photosynthetic molecules. In contrast, severe P stress in the SAB at the time of the cruise may have limited N uptake, because the deficiency of P can reduce the cell quota of this element and also might impair the uptake and decrease cell quota of N (Persson et al., 2010; Hillebrand et al., 2013; Giménez-Grau et al., 2020).

In the oligotrophic Gulf Stream region, the residing phytoplankton community has been subjected to prolonged N and P starvation, and it exhibited the lowest community-level N uptake. Picocyanobacteria are thought to have higher binding affinity for nutrients overall. Although, *Prochlorococcus,* the dominant species in this region, are able to regulate high-and low-affinity intracellular P transport systems at different P availabilities (Martiny et al., 2006; Lin et al., 2016), under the severe nutrient starvation in the Gulf Stream where both N and P (and possibly other nutrients) were likely below critical threshold values, it was not surprising to observe the lowest N uptake rates as their physiology may have already attuned to the nutrient-poor pre-condition.

### 4.2 Phytoplankton community composition and nutrient availability

Theoretical studies, culture experiments, and *in situ* data suggest diverse nutrient uptake strategies in phytoplankton populations and communities (Litchman et al., 2007; Edwards et al., 2012; Lomas et al., 2014). The size of phytoplankton cells influences physiological rates (e.g., nutrient uptake and growth rates), biotic interactions (e.g., grazing) and behaviors (e.g., sinking speed) in the fluid environment (Barton et al., 2013b; Edwards et al., 2015). Larger cells, such as diatoms, tend to have greater maximum uptake rates on a per-cell basis (Sarthou et al., 2005; Litchman et al., 2007; Edwards et al., 2011; Lomas et al., 2014) and larger nutrient storage capacity (Grover, 1991; Stolte and Riegman, 1995; Grover, 2011; Stief et al., 2022). This allows them to quickly take up nutrient pulses (Zimmerman et al., 1987) and/or store nutrients after brief periods of high supply (Bode et al., 1997; Stief et al., 2022), prolonging saturated growth beyond the nutrient pulse (Edwards et al., 2013). This explains why large sized phytoplankton (e.g., diatoms) are favored/selected under high or pulsed nitrate supplies occurring over a relatively long (~2–30 day) periods, e.g., during upwelling events (Litchman et al., 2009). Recent studies on diatom gene expression patterns suggest diatom can rapidly assimilate newly acquired nitrate and reduce carbon requirement to support high growth rates (Lampe et al., 2019; Inomura et al., 2023), highlighting their physiological adaptation to nutrient replete and intermittently replete environments. Consistent with these findings, diatoms were the dominant group in phytoplankton assemblages at both shelf regions, especially at the depths of Chl maximum, coinciding with the top of the nitracline where nutrient diffusivity or diapycnal transport usually occurs (e.g., Hales et al., 2009).

Under nutrient-limited conditions, particularly in stratified systems, phytoplankton communities rely on reduced/regenerated forms of nitrogen for growth (Raimbault et al., 1999; Fawcett et al., 2011). Gene expression patterns under nitrogen starvation reveal a dependence on regenerated nitrogen, with NH_4_^+^ and urea transporters highly expressed (Lampe et al., 2019). Smaller phytoplankton cells exhibit higher scaled nutrient binding affinities than larger cells, allowing them to maintain positive growth rates at lower nutrient concentrations (see Figure 1 in Edwards et al., 2012). In subtropical gyres with consistent stratification and weak nutrient resupply to surface waters, smaller phytoplankton cells can draw down ambient nutrient concentrations to critically low levels where larger cells cannot survive (Barton et al., 2013b). Congruent with these observations and our hypothesis, *Prochlorococcus* was found to be the dominant species in Gulf Stream surface waters. Thriving in these “ocean deserts,” *Prochlorococcus* demonstrated the capability to deploy multiple biochemical strategies simultaneously to use urea (Moore et al., 2002; Saito et al., 2014), amino acids (e.g., Zubkov et al., 2003), and cyanate (e.g., Kamennaya et al., 2008). A recent model study integrating genomic and molecular datasets detailed the cellular-scale optimization of metabolism and physiology in *Prochlorococcus* (Casey et al., 2022).

*Synechococcus* was ubiquitous across our study region, particularly dominant at the shallower euphotic depths (Figures 11D,E). *Synechococcus* is capable of mobilizing ammonium, nitrite, nitrate, urea, cyanate, and amino acids to support their growth (Lindell et al., 1998; Moore et al., 2002; Kamennaya and Post, 2011; Muñoz-Marín et al., 2020; Sato et al., 2022). In contrast to *Prochlorococcus* who lacks genes for nitrate uptake and reduction (Moore et al., 2002), the ability to assimilate both oxidized and reduced N may reflect their higher cellular N requirements, especially given the large, N-rich light-harvesting protein complexes (phycobilisomes) that must be maintained (Scanlan, 2003). This could explain the predominance of *Synechococcus* shallower in the water column (Figures 11D,E) as the low light levels at the base of the euphotic zone may not yield sufficient energy to reduce oxidized N forms (Van Oostende et al., 2017) and transport N and P into outer membrane (Kamennaya et al., 2020). It also implies a trade-off or trait that helps to explain their broader geographical distribution in waters receiving higher irradiance, regardless of nutrient conditions (Partensky et al., 1999).

Haptophytes (Type 8) were the dominant group of pico-eukaryotes (Figure 11) and comprised nearly 50% of the total Chl *a* at the depth of Chl maximum in the Gulf Stream (Figure 11F). This pattern is consistent with observations from oligotrophic areas where haptophytes (Type 8) occupied niches characterized by elevated mean nitrate and phosphate concentrations but low mean temperature and irradiance (Xiao et al., 2018). Studies also noted that diatoms and haptophytes competitively interact in natural environments (Alexander et al., 2015b). Ecologically, diatoms are generally r-strategists, characterized by rapid growth rates under suitable conditions. In contrast, haptophytes are likely K-strategists, characterized by slow growth rates (Endo et al., 2018). This distinction is further supported by a metatranscriptomic study in an oligotrophic ocean (Alexander et al., 2015b), where diatoms increased growth-related transcriptional activity under nutrient-rich conditions and haptophytes decreased. Our findings corroborate this pattern, with low diatom but high haptophyte abundances (Type 8) at the depth of the Chl maximum in the Gulf Stream (Figure 11F).

### 4.3 Correlations between N uptake rates and environmental factors

The influence of light, temperature, nutrient availability, and their interactions on primary producers are critical factors in shaping phytoplankton communities and associated biogeochemical processes (MacIsaac and Dugdale, 1972; Edwards et al., 2015, 2016; Maguer et al., 2015). In general, the North Atlantic region experiences N limitation (Graziano et al., 1996). Several studies have reported uptake rates for various nitrogen compounds, including NO_3_^−^ (Elskens et al., 1997; Rees et al., 2006; Painter et al., 2008b), urea (e.g., Painter et al., 2008a), NH_4_^+^ (Rees et al., 1999; Donald et al., 2001; Rees et al., 2006), and NO_2_^−^/amino acids (e.g., Fuhrman, 1987; Filippino et al., 2011), across a wide space of Atlantic Ocean. In addition, through WOCE and JGOFS expeditions, Harrison et al. (1996) examined N uptake kinetics across the northern Atlantic and found the uptake of NO_3_^−^ and NH_4_^+^ by phytoplankton were both concentration-and temperature-dependent. Reay et al. (1999) found that phytoplankton’s affinity for NO_3_^−^ was indeed strongly dependent on temperature and consistently decreased at temperatures below their optimum temperature, however, the affinity for NH_4_^+^ showed no clear temperature dependence. Subsequently, Smith et al. (2009) suggested that nutrient uptake patterns of phytoplankton are better explained as a trade-off between uptake capacity and affinity, and that phytoplankton cells acclimate to varying nutrient concentrations, modifying their apparent half-saturation constants for nutrient uptake. It is now understood that the uptake rates of nutrients is a manifestation of phytoplankton physiological state and demonstrate a degree of plasticity (Kwon et al., 2022).

Using both pair-wise correlations (Heatmap) and multivariate statistical analysis (redundancy analysis, RDA), we found that both temperature and salinity exhibited negative correlations with total N uptake and uptake rates of individual N compounds, whereas PN and Chl concentrations were positively correlated with total N uptake and uptake rates of individual N compounds (Figure 12). There was no significant relationship between NO_3_^−^ concentrations and total N uptake or between NO_3_^−^ concentrations and the uptake rates of other N compounds in the study area. PO_4_ concentrations showed positive correlations with the total N uptake and the uptake of individual N compounds, supporting the earlier suggestion that elevated P concentrations contribute to higher N uptake rates through synergistic effect or N-P co-limitation. In addition, distinct patterns were observed between phytoplankton species compositions and environmental factors. For example, there were negative correlations between temperature and salinity and the abundances of diatoms and pico-eukaryotes, but positive correlations between temperature and salinity and *Prochlorococcus* and *Synechococcus* fractions, corroborating what we know about these groups’ physiological preferences. *Synechococcus* dominance also showed (weak) positive correlations with cyanate uptake rates, highlighting this organisms capability to use cyanate as a nitrogen source, a pattern also observed in the North Pacific subtropical gyre (Sato et al., 2022).

**Figure 13:**
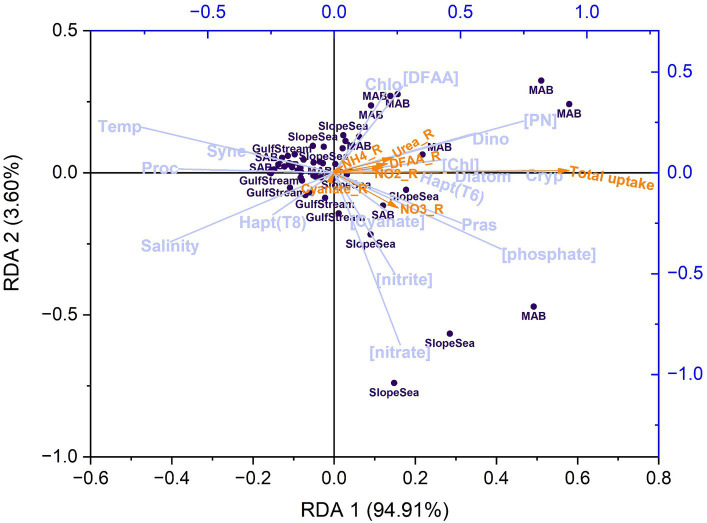
Redundancy analysis (RDA) plot showing the correlations between response variables (N uptake rates, indicated by orange arrows) and a set of explanatory variables (lavender vectors), including temperature (Temp), salinity, ambient nutrient concentrations ([phosphate], [nitrate], [nitrite], [ammonium], and [DFAA]), Chl and PN concentrations ([Chl], [PN]), and phytoplankton community composition including diatoms (Diatom), dinoflagellates (Dino), haptophytes [Type 8; Hapt(T8)], haptophytes [Type 6; Hapt(T6)], chlorophytes (Chlo), cryptophytes (Cryp), Prochlorococcus (Proc), Synechococcus (Syne), and prasinophytes (Pras). The dark purple dots indicate observations (a total of 56 after excluding data with NaNs). Note that urea was not included due to undetectable concentrations, and ammonium was not shown in the plot due to its small vector size. Total uptake, NO_3__R, NO_2__R, NH_4__R, Urea_R, DFAA_R, Cyanate_R stands for the uptake rate of total nitrogen, nitrate, nitrite, ammonium, urea, amino acids, and cyanate, respectively. DFAA stands for dissolved free amino acids.

In the RDA plot (Figure 13), the first RDA axis (RDA 1) captured nearly 95% of the variability related to the explanatory variables. The coefficient of RDA 1 for total N uptake was 0.93. Temperature, salinity, and *Prochlorococcus* exhibited the highest negative correlations (*r* < −0.6) with total N uptake, and this was particularly pronounced in observations made in the Gulf Stream. As discussed in section 4.2, elevated temperatures can lead to strong stratification, limiting nutrient resupply and availability. *Prochlorococcus* is well adapted to nutrient-poor environments, exhibits inherently low nitrogen uptake rates.

PN, phosphate, dinoflagellates, prasinophytes, and cryptophytes demonstrated the highest positive correlations (*r* > 0.5) with total N uptake. It remains unclear why dinoflagellates and cryptophytes exhibited stronger correlation with N uptake than diatoms, despite not being the dominant species. This analysis reinforces the notion that phosphorus serves as the secondary or co-limiting factor, particularly in the SAB and MAB. For individual N uptake rates, ammonium and cyanate uptake rates showed negligible correlations with any measured variables (although absolute cyanate uptake rates were ubiquity low). Correlations with the remaining individual N uptake rates were obvious but insignificant, further indicating that uptake processes are complex and influenced by phytoplankton physiological status, nutrient pre-conditioning, and the relative rates of nutrient resupply.

## 5 Conclusion

This study examined the spatial distribution of summertime phytoplankton communities and N uptake rates across a large geographical area with extremely heterogenous physical and chemical structure of the water column. The dynamic nature of the marine environment shapes the structure of the phytoplankton community, selecting for phytoplankton with physiological traits amenable to the biogeochemical conditions of the environment, who then reshape the biogeochemical environment. As hypothesized, we observed that diatoms were more abundant and occupied niches in coastal and offshore waters in the MAB and SAB. In contrast, *Prochlorococcus* was dominant in the Gulf Stream, likely due to nutrient impoverishment and light availability. Higher concentrations of inorganic phosphorus were linked to higher total N uptake rates in the MAB, congruent with observations that N is the primary limiting nutrient in the North Atlantic region, and phosphorus is the secondary or co-limiting nutrient. Nitrate and urea uptake rates together contributed ~50% of the total community N uptake. Cyanate uptake rates were low across the study region but could be up to 11% of the total N uptake at some stations within Gulf Stream. The N uptake rates, and hydrographic and biogeochemical measurements made here will help parameterize biogeochemical models for this region.

## Data availability statement

The datasets presented in this study can be found in online repositories. The CTD, nutrient, and PNPC and nitrogen uptake rates data can be accessed via https://www.bco-dmo.org/project/683923. The phytoplankton pigment data are available at https://seabass.gsfc.nasa.gov/cruise/cyanate2016.

## Author contributions

YZ: Writing – original draft, Visualization, Formal analysis, Data curation, Conceptualization. MM: Writing – review & editing, Supervision, Resources, Funding acquisition, Conceptualization. PB: Data curation, Resources, Writing – review & editing, Project administration, Methodology, Investigation. AN: Writing – review & editing, Methodology. BW: Writing – review & editing, Methodology, Investigation. AT: Writing – review & editing, Methodology, Investigation. ME: Writing – review & editing, Methodology, Investigation.

## Footnotes

^1^https://www.aviso.altimetry.fr/en/home.html
