# Supplementary material for: Nitrogen uptake rates and phytoplankton composition across contrasting North Atlantic Ocean coastal regimes north and south of Cape Hatteras
Source: Front Microbiol. 2024 May 9;15:1380179. doi: 10.3389/fmicb.2024.1380179 (PMC11113559; doi:10.3389/fmicb.2024.1380179)
Supplement: Supplementary file 1 [file Data_Sheet_1.docx]

**
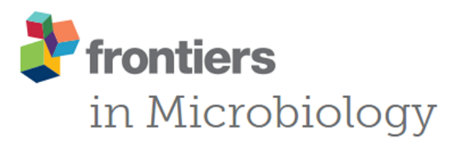
**

*Journal of Frontiers in Microbiology: Section of Aquatic Microbiology*

*Supporting Information for*

**Nitrogen uptake rates and phytoplankton composition across contrasting North Atlantic Ocean coastal regimes north and south of Cape Hatteras**

**Yifan Zhu^1, 2,^ ∗, Margaret R. Mulholland^1,^ ∗, Peter Bernhardt^1^, Aimee Renee Neeley^2^, Brittany Widner^1^, Alfonso Macías Tapia^1, 4^, and Michael A. Echevarria^1^**

*1Department of Ocean and Earth Sciences, Old Dominion University, 23529, Norfolk, Virginia, United States*

*2Department of Marine Sciences, University of Connecticut, 06340, Groton, Connecticut, United States*

*3NASA Goddard Space Flight Center, 20771, Greenbelt, Maryland, United States*

*4Office of Education, National Oceanic and Atmospheric Administration, 20910, Silver Spring, Maryland, United States*

**Correspondence:*

Yifan Zhu, [yifan.zhu@uconn.edu](mailto:yifan.zhu@uconn.edu%20)

Margaret R Mulholland, [mm](mailto:mmulholl@odu.edu)[ulholl@odu.edu](mailto:ulholl@odu.edu)

**Contents**

| Tables S1 | Figures S1 to S4 |
| --- | --- |

**Introduction**

Table S1 provides information on the diagnostic pigments used to identify phytoplankton composition.

Figures S1 to S4 provide additional results for the main text, including vertical profiles of biogeochemical parameters at individual stations and vertical and spatial distribution of specific N uptake rates.

**Table S1**. The thirteen diagnostic pigments and their affiliation to phytoplankton groups are marked with dots

| Pigments | Abbr. | Dinoflagellates  Dino | Diatoms  Diat | Haptophytes  (Type 8)  Hapt_8 | Haptophytes  (Type 6)  Hapt_6 | Chlorophytes Chlo | Cryptophytes  Cryp | *Prochlorococcus*  Proc | *Synechococcus*  Syne | Prasinophytes Pras |
| --- | --- | --- | --- | --- | --- | --- | --- | --- | --- | --- |
| Monovinyl  chlorophyll a | Chl *a* |  |  |  |  |  |  |  |  |  |
| Divinyl chlorophyll a | DV-  Chl *a* |  |  |  |  |  |  |  |  |  |
| Monovinyl  chlorophyll b | Chl *b* |  |  |  |  |  |  |  |  |  |
| Alloxanthin | Allo |  |  |  |  |  |  |  |  |  |
| 19′-Butanoyloxy  -fucoxanthin | But |  |  |  |  |  |  |  |  |  |
| Fucoxanthin | Fuco |  |  |  |  |  |  |  |  |  |
| 19′-Hexanoyloxy -fucoxanthin | Hex |  |  |  |  |  |  |  |  |  |
| Lutein | Lut |  |  |  |  |  |  |  |  |  |
| Neoxanthin | Neo |  |  |  |  |  |  |  |  |  |
| Peridinin | Peri |  |  |  |  |  |  |  |  |  |
| Prasinoxanthin | Prasino |  |  |  |  |  |  |  |  |  |
| Violaxanthin | Viol |  |  |  |  |  |  |  |  |  |
| Zeaxanthin | Zea |  |  |  |  |  |  |  |  |  |





**Figure S1.** Depth profiles of Chl, phosphate (PO_4_), nitrate (NO_3_^-^), nitrite (NO_2_^-^), ammonium (NH_4_^+^), and cyanate at selected stations.




**Figure S2.** Depth profiles of Chl, phosphate (PO_4_), nitrate (NO_3_^-^), nitrite (NO_2_^-^), ammonium (NH_4_^+^), and cyanate at selected stations.


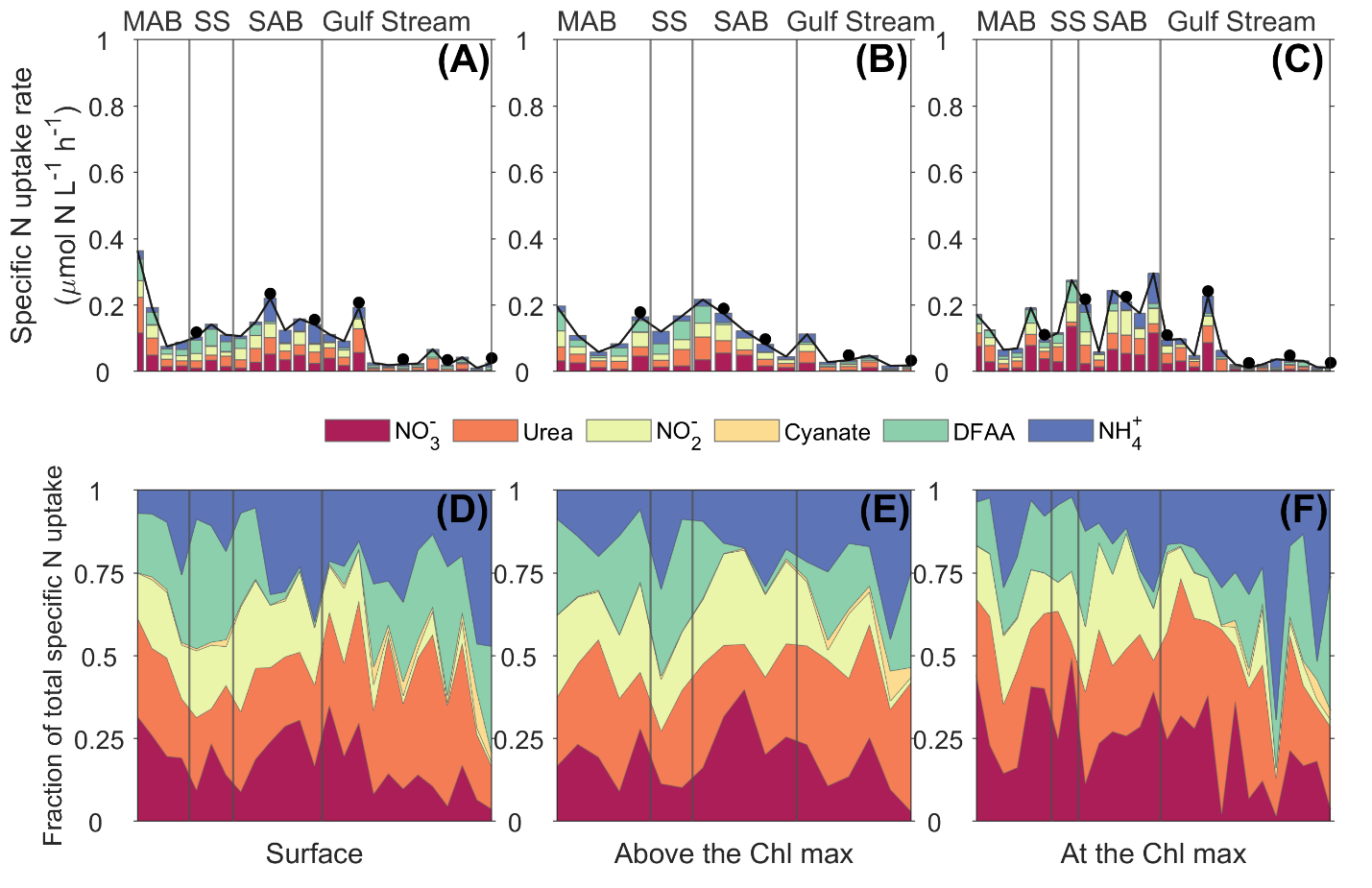


**Figure S3.** Plots of (**A−C**) specific N uptake rates, (**D−F**) the fraction of total specific N uptake contributed by the various N species measured across different regions which delimited by gray lines. MAB, SS, SAB stand for Mid Atlantic Bight, Slope Sea, and South Atlantic Bight, respectively. The left, middle, and right panels represent data from surface, above the Chl maximum, and Chl maximum depths, respectively. Black dots indicate that incubation was conducted at nighttime.


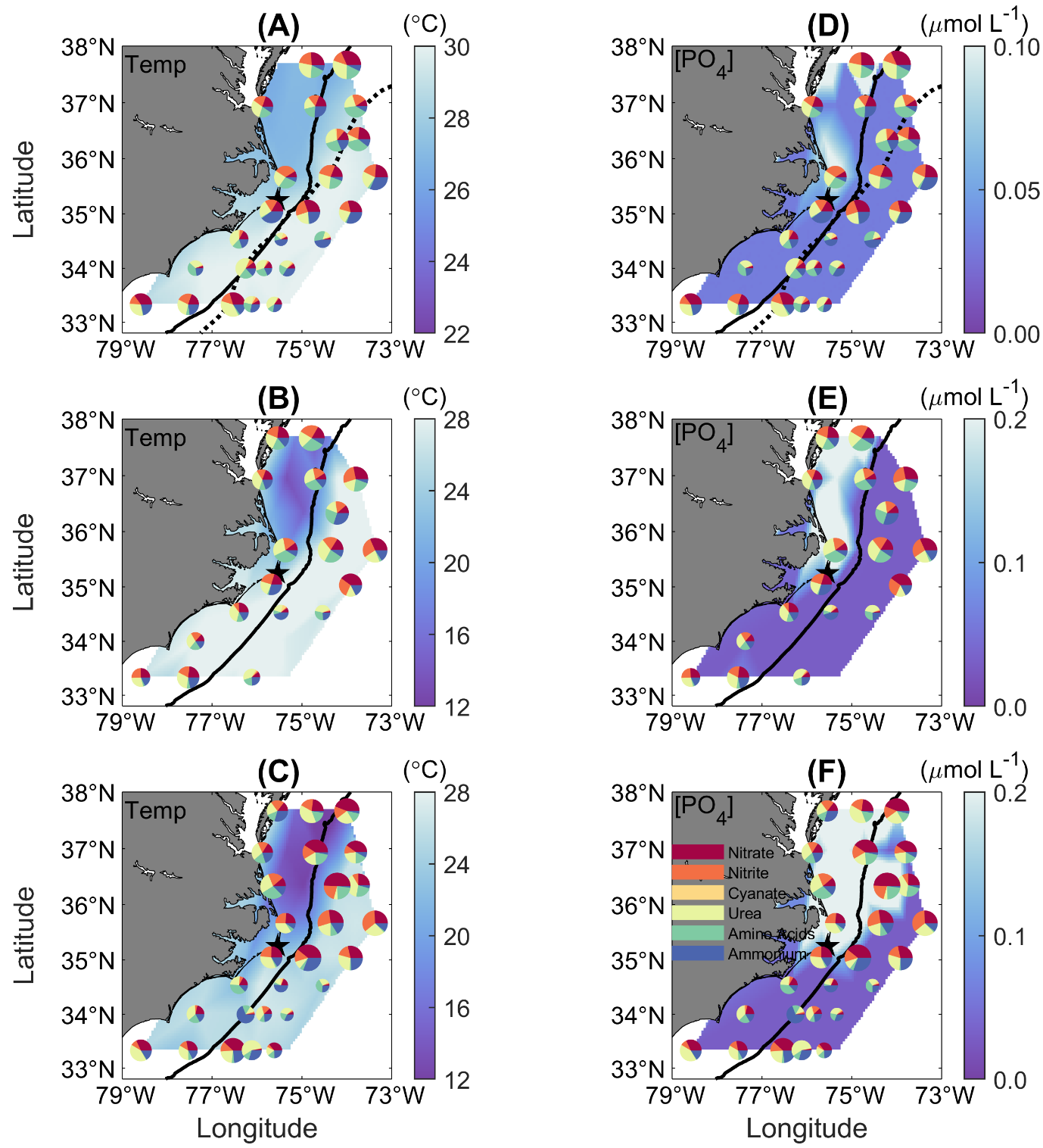


**Figure S4.** Spatial distribution of specific N uptake rates contributed by the six different N species, superimposed with (**A−C**) *in-situ* temperature and (**D−F**) PO_4_ concentrations ([PO_4_]) measured at the surface (upper panels), above the Chl maximum (middle panels), and at the Chl maximum depth (bottom panels). The size of each pie in the figure represents the total specific uptake rates, and each colored slice corresponds to one of the six tested N species. The color legend is provided in panel (**F**). Contrasts in temperature and PO_4_ concentrations among the Mid-Atlantic Bight (MAB), South Atlantic Bight (SAB), Slope Sea, and Gulf stream regions are observed. The thick black solid line in all panels mark the 200-m isobaths. The black dotted lines in upper panels represent the Gulf Stream Edge.
